# Organization of Coloration in Nemerteans: Insights from Light and Electron Microscopy

**DOI:** 10.3390/biology15171492

**Published:** 2026-09-02

**Authors:** Alexandra O. Pereverzeva, Timur Yu. Magarlamov

**Affiliations:** A.V. Zhirmunsky National Scientific Center of Marine Biology, Far Eastern Branch, Russian Academy of Sciences, 690041 Vladivostok, Russia

**Keywords:** Nemertea, pigment system, chromatophore, leucophore, mithochondria, pigment maturation

## Abstract

Although pigment systems have been extensively studied in vertebrates and cephalopods, those of many invertebrate groups, including nemerteans (Nemertea), remain poorly understood. Nevertheless, nemerteans represent a valuable model for evolutionary studies. Here, we investigated the tissue and cellular organization underlying body coloration in nine nemertean species from three classes (Palaeonemertea, Hoplonemertea, and Pilidiophora) using light and transmission electron microscopy. Our results show that coloration in nemerteans is mediated not only by specialized pigment cells (chromatophores) but also by ciliated and glandular epithelial cells. The distribution of pigment-containing structures varies considerably among taxa, with the greatest diversity observed in heteronemerteans. In *Tubulanus punctatus* and *Kulikovia torquata*, pigment is localized in ciliated epithelial cells, where pigment granules form from modified mitochondria. In contrast, in chromatophores, granule formation involves the endoplasmic reticulum or Golgi apparatus. Leucophores, responsible for white spot formation in *Kulikovia* species, are identified in nemerteans for the first time. These findings reveal high evolutionary lability of nemertean pigment systems and expand our understanding of cellular diversity in spiralian pigmentation mechanisms. This study fills a significant gap in comparative chromatophore cytology and provides a foundation for future investigations into the chemical nature and physiological functions of nemertean pigments.

## Simple Summary

Nemerteans, or ribbon worms, are marine invertebrates, many of which exhibit bright and diverse coloration. However, the mechanisms underlying their coloration remain poorly understood. In this study, we investigated the cellular and tissue organization of body coloration in nine nemertean species from three classes using light and transmission electron microscopy. We found that nemertean coloration involves several cell types, including specialized pigment cells, glandular cells, and ciliated epithelial cells. Pigment localization varies among species, occurring in different layers of the integument and even in the musculature of the body wall. Notably, we demonstrate for the first time that mitochondria serve as the site of pigment granule formation in two species from different classes, a mechanism not previously described in animals. These results advance our understanding of the cellular basis of coloration in invertebrates and provide new insights into the evolution of animal pigment systems.

## 1. Introduction

Animal pigment systems have important protective, physiological, and signaling functions, but their cellular organization remains poorly understood in many invertebrate groups, including Nemertea. Nemerteans occupy an important phylogenetic position within Spiralia, making them useful for comparative studies of pigment system evolution. According to modern molecular phylogenetic data, nemerteans are placed within Lophotrochozoa, often as a sister lineage to Trochozoa or within a broader clade with Lophophorata [1,2].

Many nemerteans exhibit bright coloration, which often serves as an important species-specific character, particularly in taxa with bicolored or multicolored patterns. The mechanisms underlying this coloration were first examined in naturalistic studies of the late 19th and early 20th centuries. These works showed that, across different nemertean classes, coloration is associated with distinct structures localized in different layers of the body wall. In heteronemerteans, pigment is located within the cutis and musculature and is represented by small granules [3,4,5,6]. However, in *Cerebratulus marginatus*, the primary body coloration is determined by pigmented mucoid glandular cells [6]. In the hoplonemertean *Drepanophorus serraticollis*, pigment is located in the basal region of the epidermis [4]. In the palaeonemertean *Carinella polymorpha* (=*Tubulanus polymorphus*), it occurs as small granules within ciliated cells, forming a dense layer beneath the ciliary apparatus [5,6]. More recent studies on the ultrastructural organization of pigment cells are limited to three works and have been conducted on heteronemerteans *Lineus atrocaeruleus* [7], *Lineus sanguinius* [7] and *Micrura bella* [8]. According to these studies, pigment cells are located within the cutis and occur in the subepidermal musculature, and exhibit stellate, dendritic, or amoeboid morphologies. Pigment granules of varying ultrastructural organization have been described within these cells.

In the present study, we investigated pigment localization at both the light microscopic and ultrastructural levels in nine nemertean species representing three classes. The aim was to clarify the tissue and cellular basis of body coloration in nemerteans. We show that coloration is not produced by a single cell type but instead results from a combination of cellular elements, including both specialized pigment cells and cells for which pigment formation is not a primary function. These findings fill an important gap in comparative chromatophore cytology and provide new insights into evolutionary transitions between pigment system types, as well as the influence of phylogenetic position on pigment system organization and regulation.

## 2. Materials and Methods

Nemerteans *Tubulanus punctatus* Takakura, 1898 (Palaeonemertea: Tubulaniformes) (*n* = 2), *Tetrastemma stimpsoni* Chernyshev, 1992 (Hoplonemertea: Monostilifera) (*n* = 3), *Oerstedia oculata* Kulikova, 1987 (Hoplonemertea: Monostilifera) (*n* = 2), *Micrura bella* Stimpson, 1857 (*n* = 2), *Kulikovia alborostrata* Takakura, 1898 (*n* = 5), *K. manchenkoi* Chernyshev, Polyakova, Turanov & Kajihara, 2017 (*n* = 4), and *K. torquata* Coe, 1901 (*n* = 2) (all Pilidiophora: Heteronemertea) were collected from the rhizoids of the brown alga *Saccharina* sp. in Spokoynaya Bay, Sea of Japan, during July–August 2020–2025. Nemerteans *Lineus ruber* (Müller, 1774) (*n* = 2) and *L. viridis* (Müller, 1774) (*n* = 2) (all Pilidiophora: Heteronemertea) were collected from the littoral zone of the Velikaya Salma Strait, Kandalaksha Gulf, White Sea, in August 2025. The nemertean *Baseodiscus hemprichii* (Ehrenberg, 1831) (Pilidiophora: Heteronemertea) (*n* = 1) was collected from the Andaman Sea and identified by A.V. Chernyshev. Nemerteans were maintained in aquaria with seawater at 4 °C. Prior to fixation, the worms were placed in a 7% magnesium chloride solution until complete muscle relaxation was achieved.

Molecular identification of species was carried out using the cytochrome c oxidase subunit I (COI) gene. A detailed protocol was published by Chernyshev and Kuznetsov [9]. Briefly, DNA was extracted using DNAzol reagent (Thermo Fisher Scientific, Waltham, MA, USA), and the COI gene was amplified using GoTaq^®^ Master Mix 2× (Promega, Madison, WI, USA) with the primers LCO1490 (5′-GGTCAACAAATCATAAAGATATTGG) and HCO2198 (5′-TAAACTTCAGGGTGACCAAAAAATCA) [10]. This was followed by PCR product purification and Sanger sequencing on an ABI Prism 3500 Genetic Analyzer (Applied Biosystems, Waltham, MA, USA). The resulting sequences were deposited in Genbank under the accession numbers listed in Table 1.

To obtain unstained frozen sections, nemerteans were fixed in 4% paraformaldehyde in PBS for 1 h, washed three times in PBS (0.1 M, pH = 7.4) for 30 min at room temperature, and then transferred to 20% sucrose. Cryosections were prepared from the foregut region. Samples were embedded in tissue freezing medium (Leica Biosystems, Nußloch, Germany) and 10 μm-thick sections were cut using a Thermo HM 560 microtome (Thermo Fisher Scientific, Waltham, MA, USA). Sections were examined using an Olympus IX83 microscope (Evident Scientific, Tokyo, Japan).

To analyze the morphological organization of the integument and for electron microscopy, 1–2 mm cross-sections from the foregut region were fixed in 2.5% glutaraldehyde (Electron Microscopy Sciences, Hatfield, PA, USA) in 0.2 M cacodylate buffer (CB) (Electron Microscopy Sciences, Hatfield, PA, USA) supplemented with 0.15 M sodium chloride for 1 h, followed by rinsing in CB. Post-fixation was carried out in 1% osmium tetroxide (Electron Microscopy Sciences, Hatfield, PA, USA) for 1 h. The material was then dehydrated through a graded ethanol–acetone series and embedded in a mixture of Epon and Araldite epoxy resins (Electron Microscopy Sciences, Hatfield, PA, USA).

For morphological analysis, transverse semithin sections (0.7 µm thick) were prepared using a Leica UC6 ultramicrotome (Leica Microsystems, Wetzlar, Germany), stained with methylene blue (Sigma-Aldrich, Burlington, MA, USA), and examined using an Olympus IX83 microscope.

For electron microscopy, ultrathin sections (60 nm thick) were prepared using a Leica UC6 ultramicrotome, contrasted with uranyl acetate and lead citrate, and examined using Libra 120 and Sigma 300 VP electron microscopes (Zeiss, Oberkochen, Germany).

## 3. Results

### 3.1. Integument Organization

**Figure 1:**
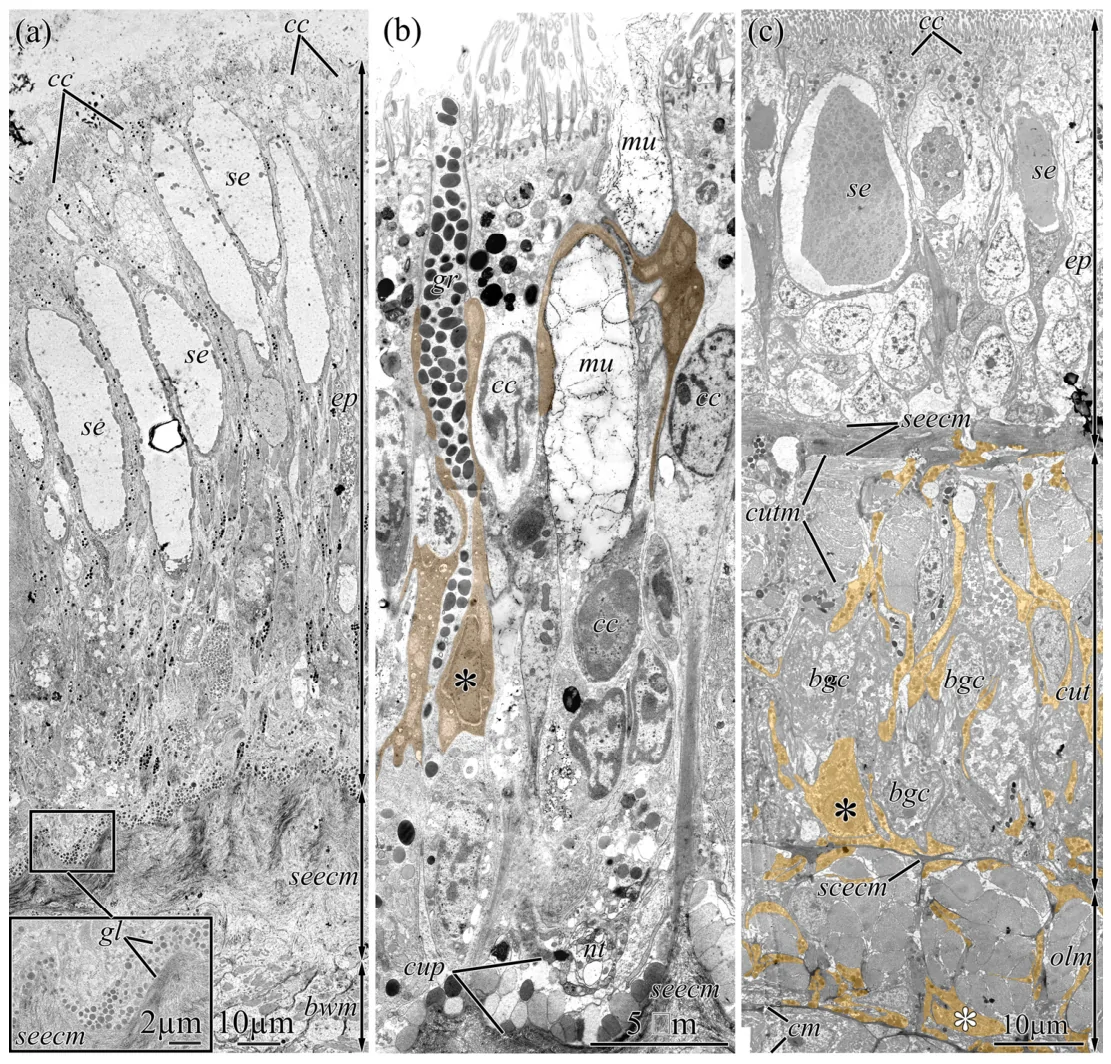
Transmission electron micrographs of transverse sections of nemertean integument types. (**a**) *Tubulanus punctatus*, panoramic view of the integument. High magnification inset shows a glia-like layer resting on the extracellular matrix. (**b**) *Oerstedia oculata*, panoramic view of the integument with the cell body of a pigment cell (asterisk) located in the middle region of the epidermis. (**c**) *Lineus viridis*, panoramic view of the integument with cell bodies of pigment cells located in the basal region of the cutis (black asterisk) and in the longitudinal musculature of the body wall (white asterisk). Abbreviations: *bgc*, cell body of cutis glandular cell; *bwm*, body wall musculature; *cc*, ciliated cell; *cm*, circular musculature; *cup*, cup zone; *cut*, cutis; *cutm*, cutis musculature; *ep*, epidermis; *gl*, glia-like layer; *gr*, granular cell; *mu*, mucous cell; *nt*, nerve trunk; *olm*, outer longitudinal musculature of the body wall; *scecm*, subcuticular extracellular matrix; *se*, serous cell; *seecm*, subepidermal extracellular matrix.

In all examined nemerteans, body coloration is primarily associated with the integument; in Heteronemertea, pigment is also present in the outer longitudinal musculature of the body wall. The studied species exhibit three types of integument organization: palaeonemertean (*T. punctatus*) (Figure 1a), hoplonemertean (*O. oculata*, *Te. stimpsoni*) (Figure 1b) and heteronemertean (*Kulikovia alborostrata*, *K. manchenkoi*, *K. torquata*, *Lineus ruber*, *L. viridis*, *B. hemprichii*) (Figure 1c). The palaeonemertean type consists of a single-layered pseudostratified ciliated epithelium (epidermis) lying on a layer of extracellular matrix (ECM) (Figure 1a). In the basal region of the epidermis, a layer of glia-like cells is present (Figure 1a inset). The hoplonemertean type comprises the epidermis, a basal “cup zone” consisting of vacuolate-like cup- shaped structures, and the ECM (Figure 1b). In heteronemerteans, the integument includes the epidermis, ECM, and cutis (Figure 1c). The cutis is a connective tissue layer located immediately beneath the ECM and contains subepidermal (cutis) glandular cells, muscle fibers, and a subepidermal nerve plexus. It is separated from the musculature of the body wall by a subcuticular ECM of variable thickness (Figure 1c).

### 3.2. Distribution of Body Pigments

#### 3.2.1. Palaeonemertea

**Figure 2:**
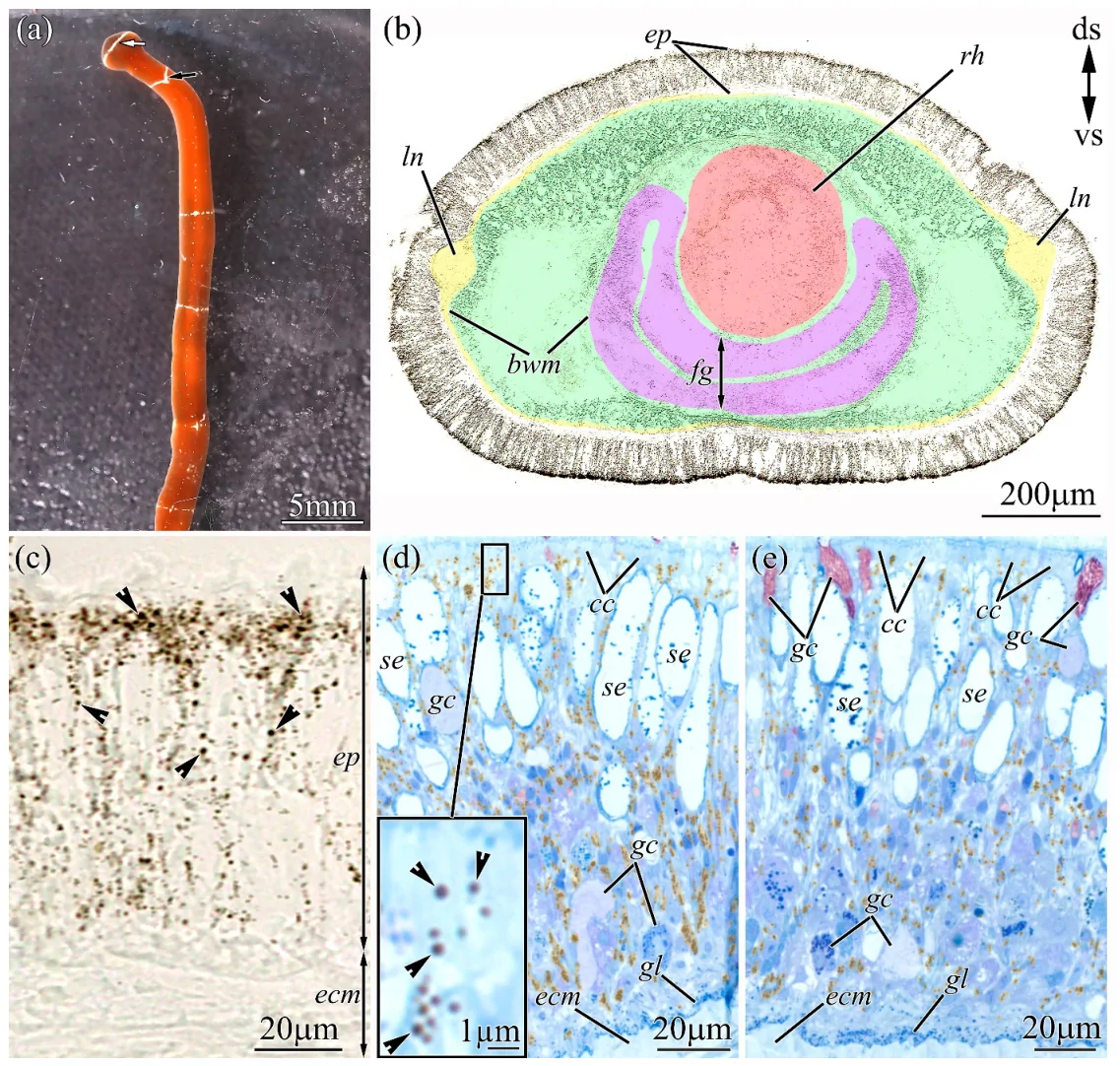
Light micrographs of a living specimen (**a**) and thick (**b**,**c**) and semithin (**d**,**e**) transverse sections of the palaeonemertean *Tubulanus punctatus*. (**a**) Live specimen. The white arrow indicates the white ring in the precerebral region; the black arrow indicates the V-shaped white ring in the oral region. (**b**) Panoramic view of the body in the foregut region. (**c**) Ventral epidermis showing brown pigment granules (arrowheads). (**d**) Dorsal epidermis showing brown pigment granules predominantly concentrated in the apical region. High-magnification inset shows the apical region of the ciliated cell with pigment granules (arrowheads). (**e**) Ventral epidermis showing uniformly distributed brown pigment granules. Abbreviations: *bwm*, body wall musculature; *cc*, ciliated cell; *ds*, dorsal side; *ecm*, extracellular matrix; *ep*, epidermis; *fg*, foregut; *gc*, glandular cell; *gl*, glia-like layer; *ln*, lateral nerve cord; *rh*, rhynchocoel; *se*, serous cell; *vs*, ventral side.

The body coloration of *T. punctatus* is brown, with the dorsal side darker than the ventral side (Figure 2a). A white ring is present on the head in the cerebral region. Posterior to the head ring, in the oral region, a second white V-shaped dorsal ring occurs, with its apex directed backward. Three thin, dotted white longitudinal stripes extend along the entire body (two lateral and one median dorsal) and are intersected by numerous white transverse rings.

The distribution of pigment in the integument of *T. punctatus* was studied in the foregut region lacking white stripes. Pigment occurs as dark brown granules (Figure 2b–e), distributed throughout the body (Figure 2b) but more concentrated on the dorsal side (Figure 2d). The granules are restricted to ciliated cells, where they are predominantly localized in the apical region (Figure 2c,d, inset).

#### 3.2.2. Hoplonemertea

**Figure 3:**
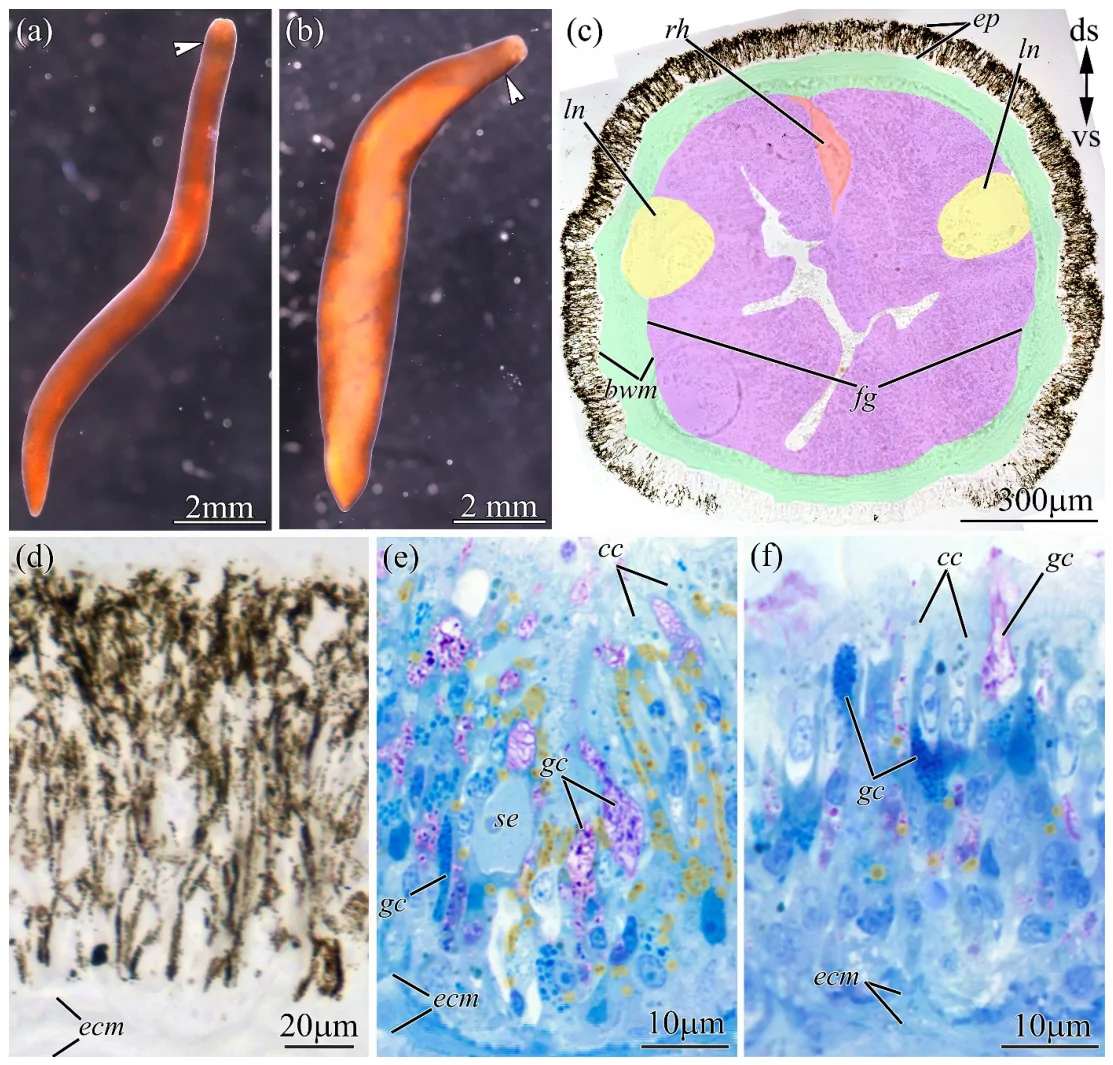
Light micrographs of living specimens (**a**,**b**) and thick (**c**) and semithin (**d**–**f**) transverse sections of the hoplonemertean *Oerstedia oculata*. (**a**) Dorsal view of a live specimen. White arrowhead indicates the head. (**b**) Ventral view of a live specimen. White arrowhead indicates the head. (**c**) Panoramic view of the body in the foregut region. (**d**) Dorsal epidermis showing evenly distributed brown pigment granules. (**e**) Dorsal epidermis showing evenly distributed brown pigment granules. (**f**) Ventral epidermis showing singly scattered brown pigment granules. Abbreviations: *bwm*, body wall musculature; *cc*, ciliated cell; *ds*, dorsal side; *ecm*, extracellular matrix; *ep*, epidermis; *fg*, foregut; *gc*, glandular cell; *ln*, lateral nerve cord; *rh*, rhynchocoel; *se*, serous cell; *vs*, ventral side.

The body coloration of *O. oculata* is uniformly brown on the dorsal and lateral sides (Figure 3a), while the ventral side lacks pigmentation (Figure 3b).

Pigment in the integument of *O. oculata* occurs as dark brown granules. These granules are concentrated predominantly on the dorsal and lateral sides of the body (Figure 3c,e), with only single granules present ventrally (Figure 3f). Pigment granules are distributed throughout the epidermis, from the apical to the basal region, and do not form distinct clusters (Figure 3d,e).

**Figure 4:**
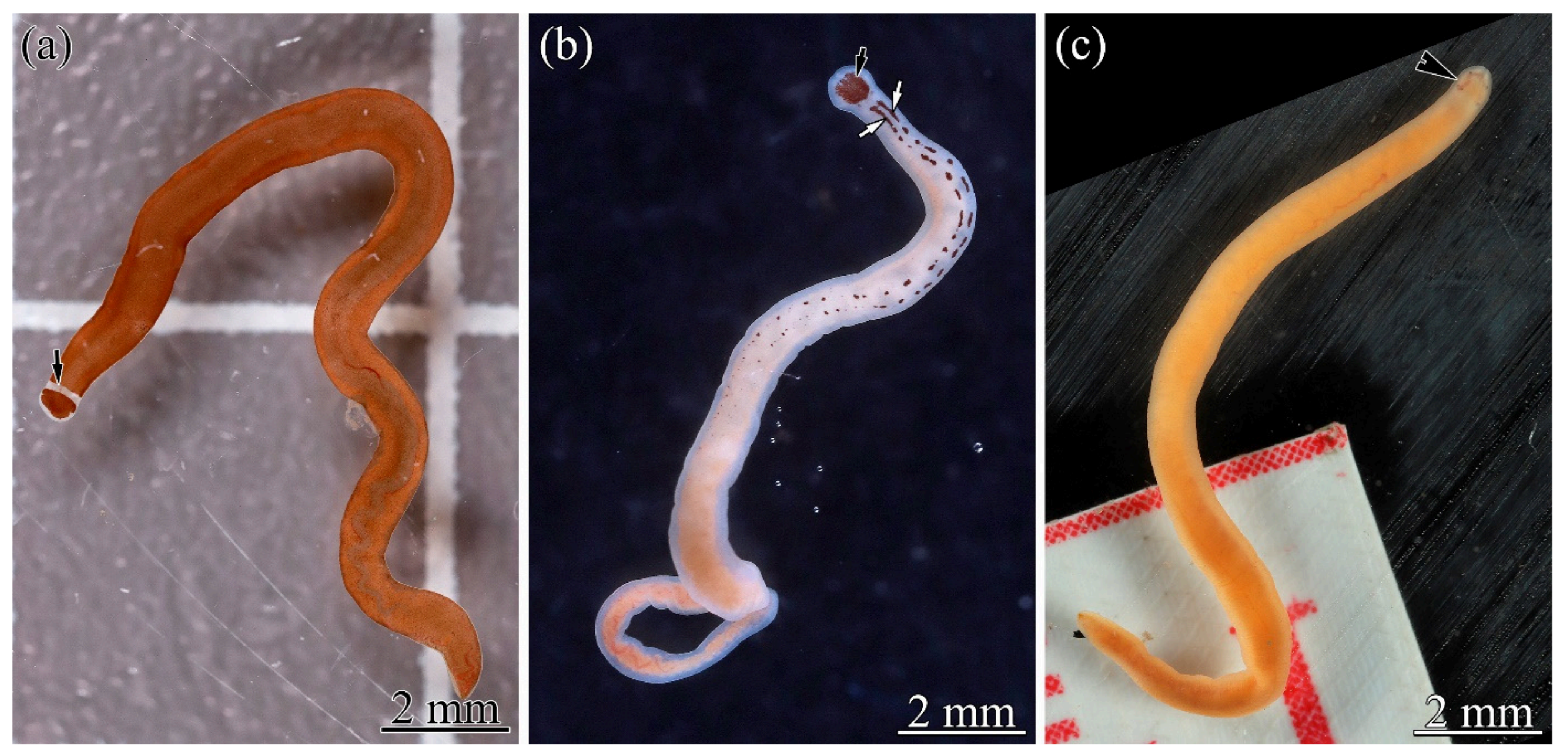
Color morphs of living specimens of *Tetrastemma stimpsoni*. (**a**) Brown color morph with a white cervical stripe (black arrow). (**b**) Morph with two interrupted dorsal stripes (white arrows) and a dark frontal spot in the cerebral region (black arrow). (**c**) Morph with pale yellow coloration. The black arrowhead indicates the head.

The body coloration of *Te. stimpsoni* is polymorphic and includes uniformly dark, spotted or striped (one to three dorsal stripes), and pale-yellow morphs (Figure 4). In all morphs, the ventral side is markedly lighter than the dorsal side. In some individuals, the head is separated from the body by a white cervical stripe (Figure 4a). A dark frontal spot is often present in the cerebral region (Figure 4b), sometimes accompanied by contrasting light areas. Intermediate color morphs were also observed.

The distribution of pigment in the integument of *Te. stimpsoni* was examined in the foregut region of three morphs: a uniformly dark morph with a white cervical stripe and a white stripe at the extreme anterior end (Figure 4a), a morph with two interrupted dark dorsal stripes (Figure 4b), and a pale-yellow morph (Figure 4c).

**Figure 5:**
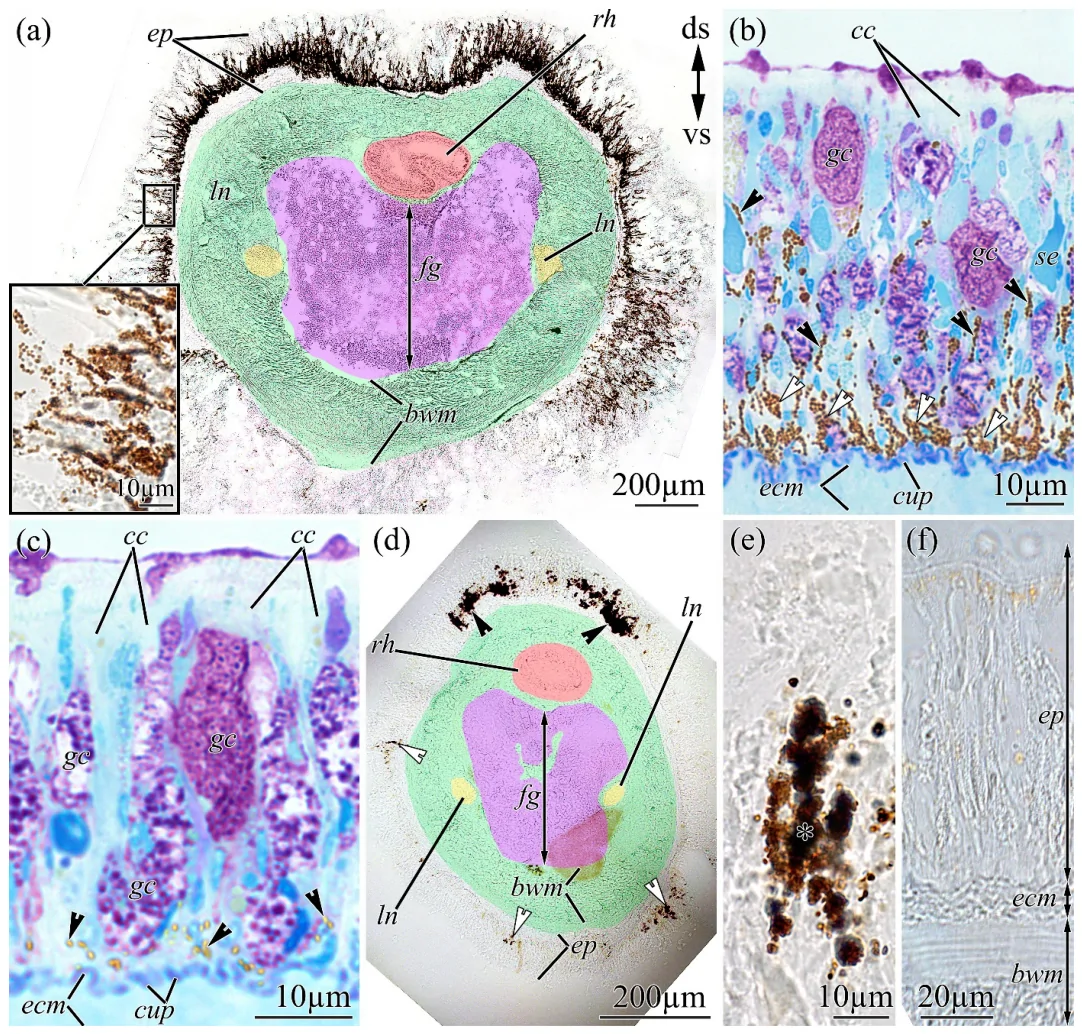
Light micrographs of thick (**a**,**d**–**f**) and semithin (**b**,**c**) transverse sections of the hoplonemertean *Tetrastemma stimpsoni*. (**a**) Panoramic view of the body in the foregut region of the uniformly dark morph. High-magnification inset shows the basal region of the epidermis containing brown pigment granules. (**b**) Dorsal epidermis of the uniformly dark morph showing brown pigment granules. In the apical and middle regions, granules form strand-like structures (black arrowheads), whereas in the basal region they form large aggregates (white arrowheads). (**c**) Ventral epidermis of the uniformly dark morph showing singly scattered brown pigment granules (arrowheads) in the basal region. (**d**) Panoramic view of the body in the foregut region of the morph with two dark dorsal stripes. Black arrowheads indicate pigment accumulations on the dorsal side; white arrowheads indicate small clusters of pigment granules on the lateral and ventral sides. (**e**) High-magnification view of a dorsal pigment spot (asterisk) in the morph with two dark dorsal stripes. (**f**) Dorsal body wall of the foregut region in the pale yellow morph. Abbreviations: *bwm*, body wall musculature; *cc*, ciliated cell; *cup*, cup zone; *ds*, dorsal side; *ecm*, extracellular matrix; *ep*, epidermis; *fg*, foregut; *gc*, glandular cell; *ln*, lateral nerve cord; *rh*, rhynchocoel; *se*, serous cell; *vs*, ventral side.

In the uniformly dark morph, pigment occurs as dark-brown granules (Figure 5a inset) and is located mainly on the dorsal and lateral sides of the body (Figure 5b), with only single granules present ventrally (Figure 5c). On the dorsal and lateral sides, pigment granules are found throughout the epidermis (Figure 5a) but are unevenly distributed, with the highest concentration in the basal region, excluding the basal cup zone (Figure 5b). In the middle and apical regions, granules are less abundant and form strands extending toward the epithelial surface without reaching it. On the ventral side, pigment granules are sparse and restricted to the basal region of the epidermis (Figure 5c).

In the morph with two dark dorsal stripes, pigment also occurs as dark-brown granules (Figure 5d), forming dense clusters in the basal region of the dorsal epidermis (Figure 5e). Small clusters are also present in the ventral and lateral epidermis.

In the pale-yellow morph, dark pigment granules were not detected (Figure 5f). However, weakly yellow-stained granules were observed in the apical region of the epidermis (Figure 5f).

#### 3.2.3. Pilidiophora: Heteronemertea

**Figure 6:**
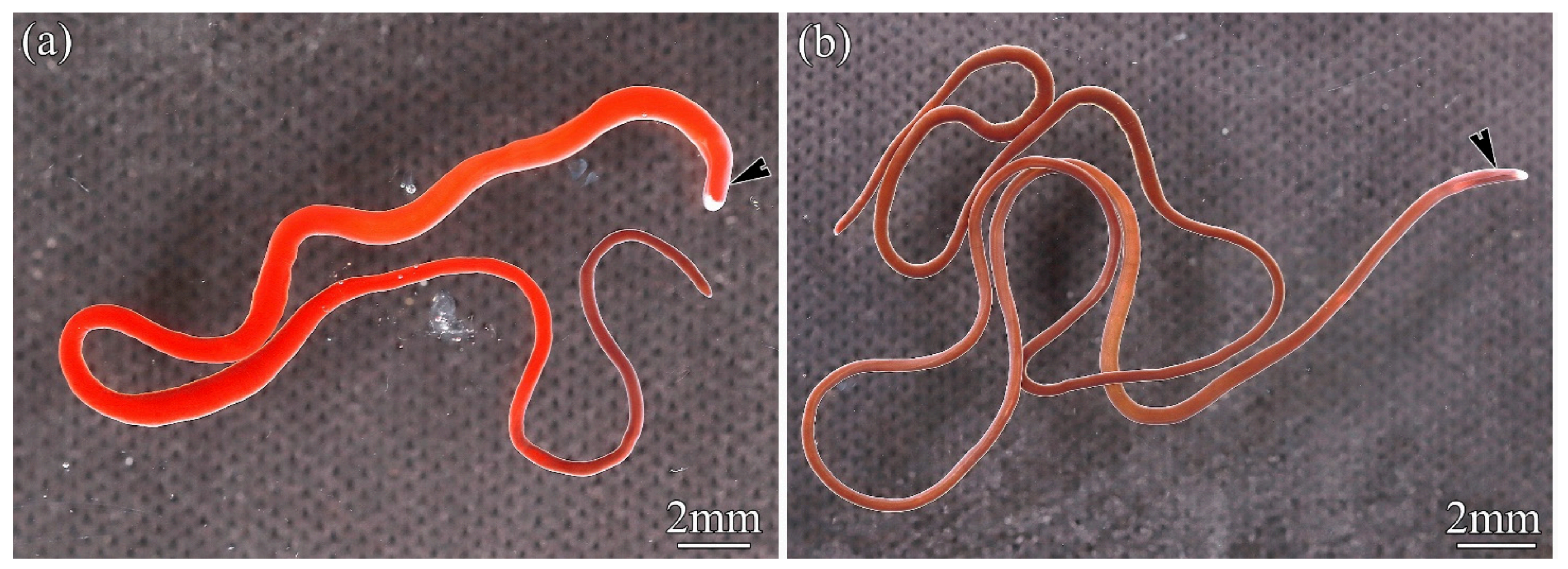
Living specimens of *Kulikovia alborostrata*. Arrowhead indicates the head. (**a**) Light morph. (**b**) Dark morph.

The body coloration of *K. alborostrata* varies from red (Figure 6a) to brown (Figure 6b), with the dorsal side slightly darker than the ventral side. A small white spot is present at the extreme anterior end of the head.

**Figure 7:**
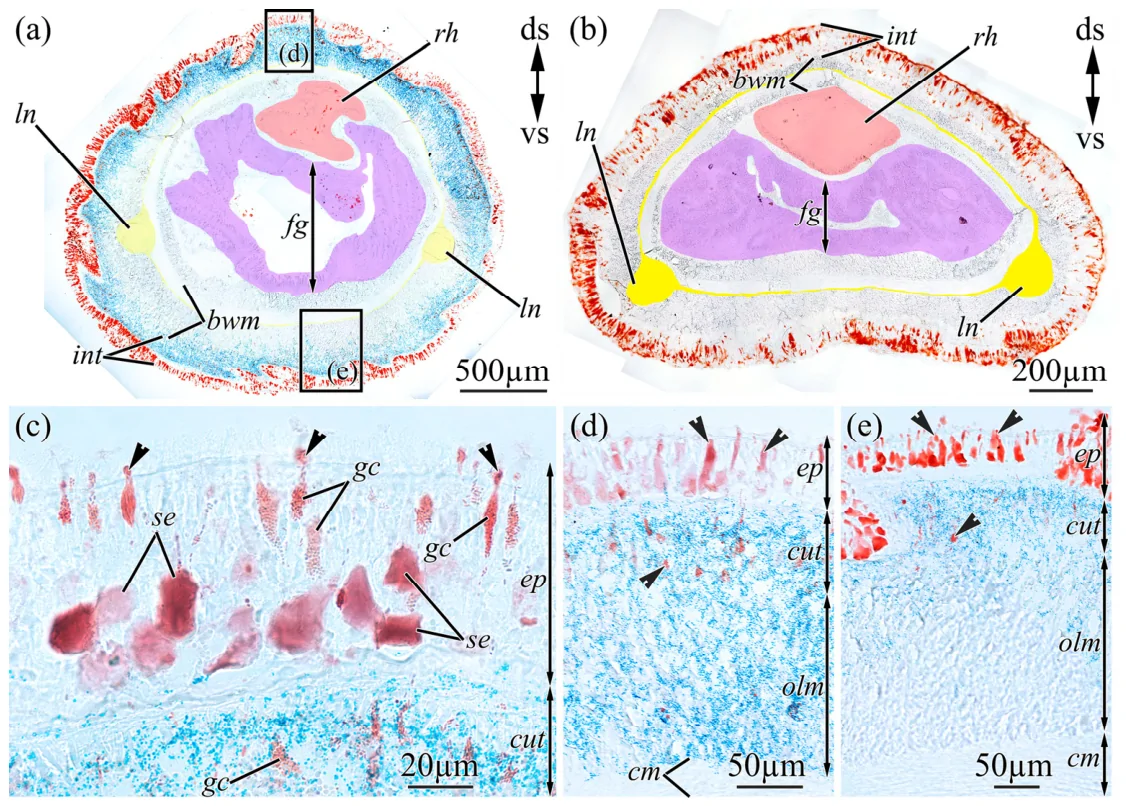
Light micrographs of thick transverse sections of the heteronemertean *Kulikovia alborostrata.* (**a**) Panoramic view of the body in the foregut region of the dark morph. (**b**) Panoramic view of the body in the foregut region of the light morph. (**c**) Dorsal integument of the dark morph. Arrowheads indicate red-colored secretion released by glandular cells onto the epidermal surface. (**d**) Dorsal body wall of the dark morph. Arrowheads indicate glandular cells containing red pigment. (**e**) Ventral body wall of the dark morph. Arrowheads indicate glandular cells containing red pigment. Abbreviations: *bwm*, body wall musculature; *cm*, circular musculature; *cut*, cutis; *ds*, dorsal side; *ep*, epidermis; *fg*, foregut; *gc*, glandular cell; *int*, integument; *ln*, lateral nerve cord; *olm*, outer longitudinal musculature of the body wall; *rh*, rhynchocoel; *se*, serous cell; *vs*, ventral side.

The distribution of pigment in the integument of *K. alborostrata* was studied in individuals with red (light morph) and brown (dark morph) coloration. The dark morph possesses two pigment types—red and blue (Figure 7a), while the light morph contains only red pigment (Figure 7b). Red pigment is evenly distributed along the dorsoventral axis and localized in epidermal serous and cutis glandular cells (Figure 7c). These cells release a colored secretion, imparting a pinkish coloration to the surrounding water (Figure 7c arrowheads). Blue pigment occurs as blue granules with an uneven dorsoventral distribution. On the dorsal side, granules are abundant, uniformly distributed, and extend to the circular musculature of the body wall (Figure 7d). On the ventral side, they are sparse and reach only half the thickness of the longitudinal musculature of the body wall (Figure 7e).

**Figure 8:**
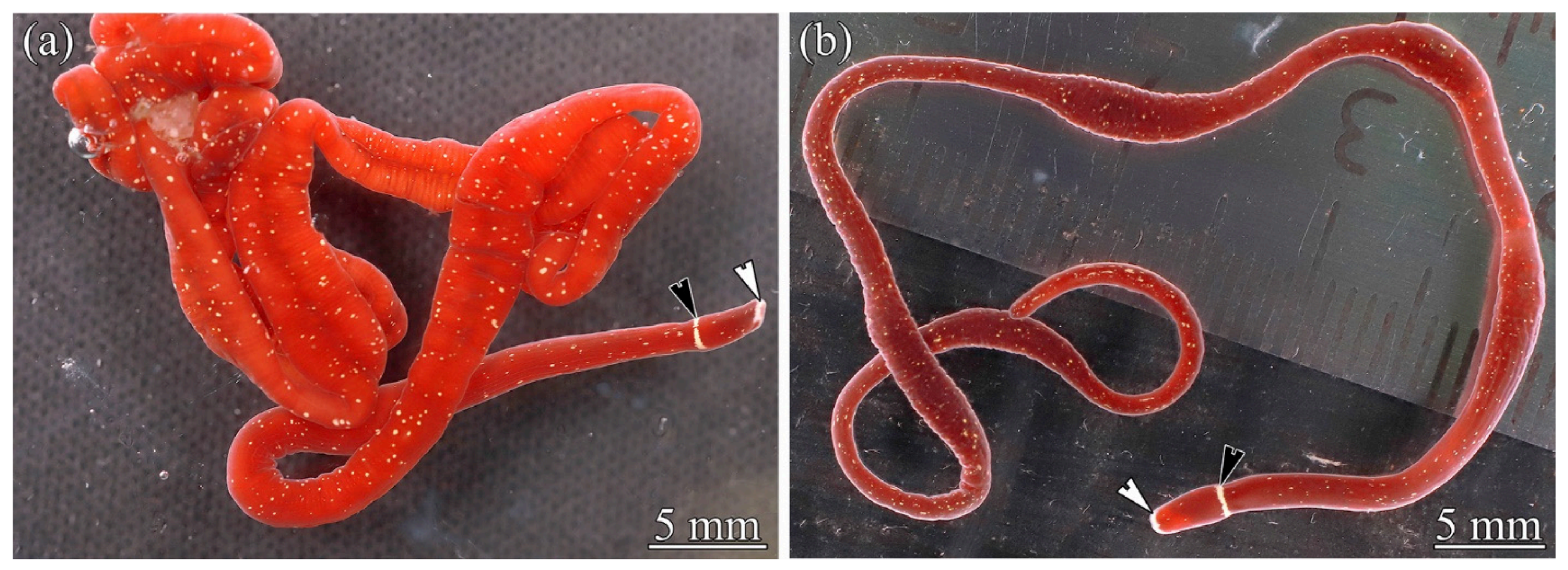
Living specimens of *Kulikovia manchenkoi*. Black arrowhead indicates the cervical stripe; white arrowhead indicates the white spot at the extreme anterior end of the head. (**a**) Light morph. (**b**) Dark morph.

The body coloration of *K. manchenkoi* ranges from cherry red (Figure 8a) to violet red (Figure 8b), with the dorsal side slightly darker than the ventral side. A small white spot is present at the extreme anterior end of the head. A white cervical stripe is located on the dorsal side in the preoral region (Figure 8a,b). The dorsal and lateral surfaces are covered with irregular white speckles.

**Figure 9:**
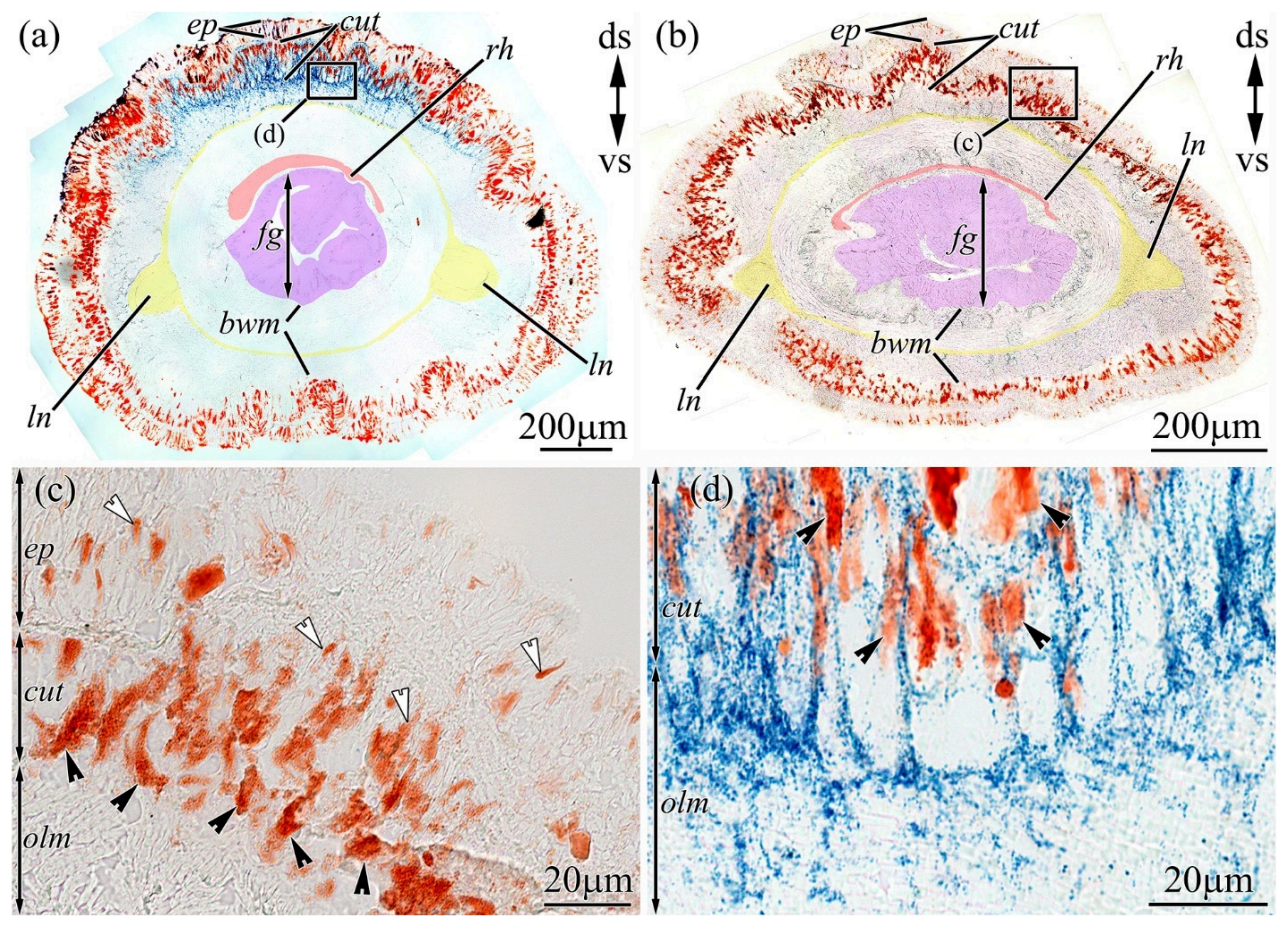
Light micrographs of thick transverse sections of the heteronemertean *Kulikovia manchenkoi*. (**a**) Panoramic view of the body in the foregut region of the dark morph. (**b**) Panoramic view of the body in the foregut region of the light morph. (**c**) Dorsal integument of the light morph showing pigment-containing cell bodies (black arrowheads) and necks (white arrowheads) of cutis glandular cells. (**d**) Proximal dorsal cutis of the dark morph showing pigment-containing cell bodies of cutis glandular cells (arrowhead). Abbreviations: *bwm*, body wall musculature; *cut*, cutis; *ds*, dorsal side; *ep*, epidermis; *fg*, foregut; *ln*, lateral nerve cord; *olm*, outer longitudinal musculature of the body wall; *rh*, rhynchocoel; *vs*, ventral side.

The distribution of pigment in the integument of *K. manchenkoi* was studied in individuals with red (light morph) and violet red (dark morph) coloration. As in *K. alborostrata*, both red and blue pigments occur, with morph-dependent presence: dark morphs contain both pigment types (Figure 9a), whereas light morphs contain only red pigment (Figure 9b). Red pigment is evenly distributed along the dorsoventral axis and is associated exclusively with epidermal and cutis glandular cells (Figure 9c). Blue pigment is restricted to the dorsal side and occurs as blue granules (Figure 9d). These granules are located mainly in the cutis, forming accumulations near the subcuticular ECM, with a smaller fraction present in the longitudinal musculature of the body wall, where they extend to the circular musculature but do not penetrate it (Figure 9a,d).

**Figure 10:**
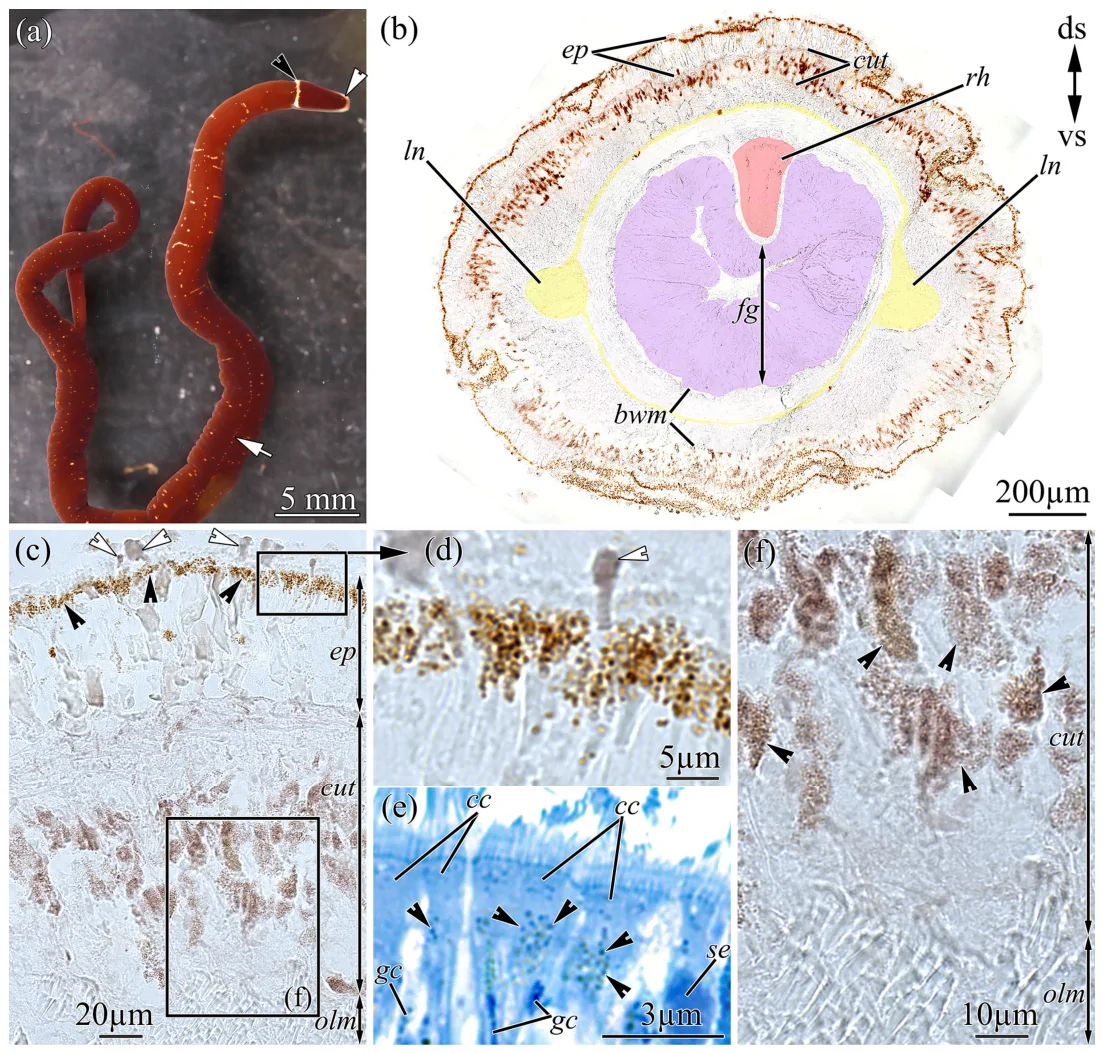
Light micrographs of a living specimen (**a**) and thick (**b**–**d**,**f**) and semithin (**e**) transverse sections of the heteronemertean *Kulikovia torquata*. (**a**) Living specimen with a white cervical stripe (black arrowhead), white spot at the extreme anterior end of the head (white arrowhead) and dorsal dotted white stripe (arrow). (**b**) Panoramic view of the body in the foregut region. (**c**) Dorsal integument showing papillae of pigment-containing glandular cells (white arrowheads) and brown granules in the apical region of the epidermis (black arrowheads). (**d**) Apical region of the epidermis showing papillae of pigment-containing glandular cells (arrowhead). (**e**) Apical region of epidermal ciliated cells showing pigment granules (arrowheads). (**f**) Distal region of the cutis showing pigment-containing glandular cells (arrowheads). Abbreviations: *bwm*, body wall musculature; *cc*, ciliated cell; *cut*, cutis; *ds*, dorsal side; *ep*, epidermis; *fg*, foregut; *gc*, glandular cell; *ln*, lateral nerve cord; *olm*, outer longitudinal musculature of the body wall; *rh*, rhynchocoel; *vs*, ventral side.

The body coloration of *K. torquata* varies from reddish brown to brown, with the dorsal side slightly darker than the ventral side (Figure 10a). A small white spot is present at the extreme anterior end of the head, and a white dorsal cervical stripe occurs in the preoral region. The dorsal and lateral surfaces are covered with irregular white speckles, some of which are elongated and stripe-like. A thin, dotted median dorsal stripe extends along the entire body (Figure 10a).

Two pigment types are present in the integument of *K. torquata*: one located in the apical region of the epidermis and the other in the cutis (Figure 10b,c), both distributed evenly along the dorsoventral axis (Figure 10b). The epidermal pigment is represented by brown granules associated with ciliated cells (Figure 10d). Within these cells, granules form dense clusters of approximately 30–50 per cross-section (Figure 10e). The cutis-associated pigment is brown to red-brown and is restricted to the cell bodies of glandular cells (Figure 10c,f). In some preparations, it is also observed in portions of the excretory ducts and papillae (Figure 10c).

**Figure 11:**
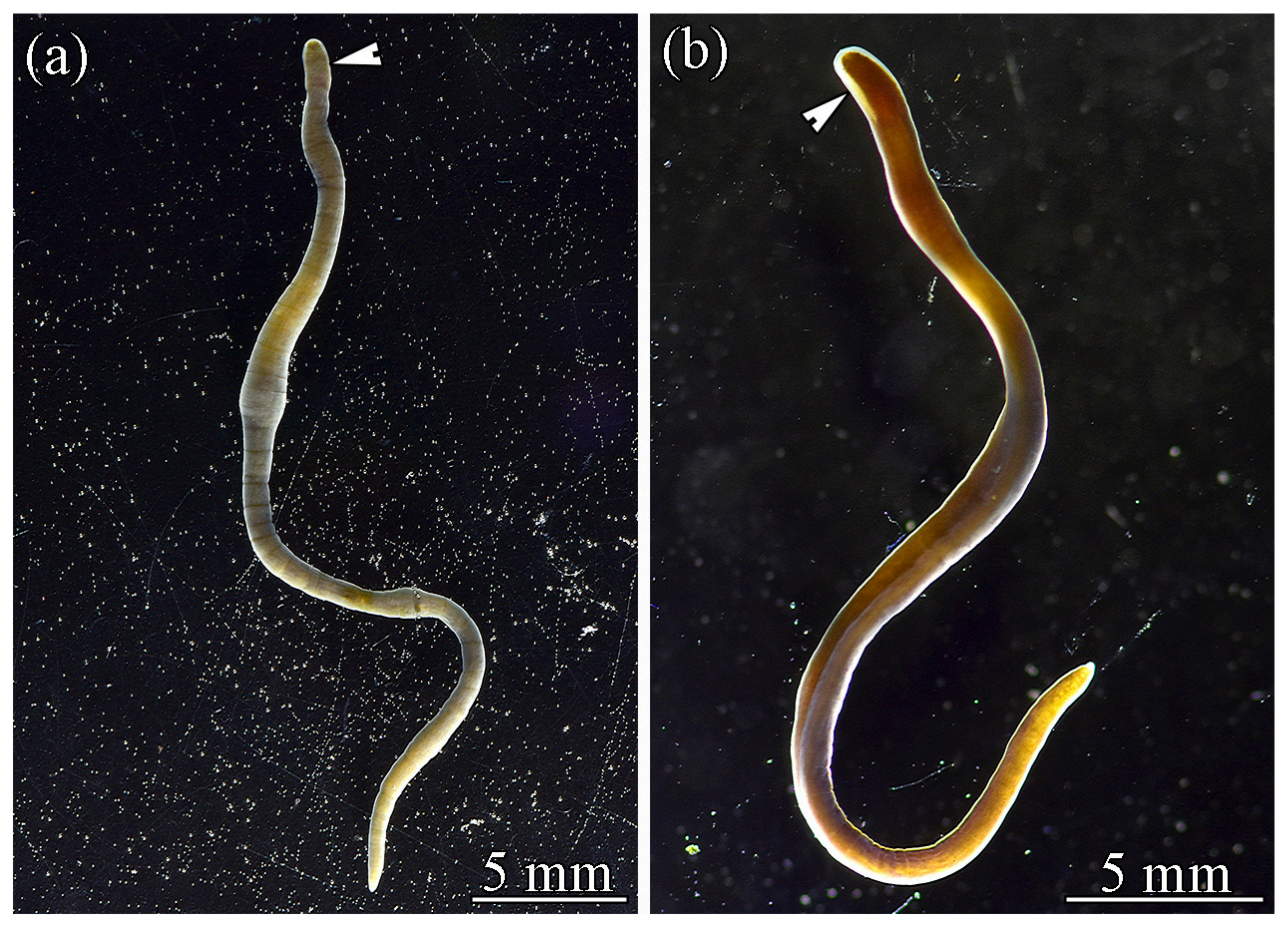
Living specimens of *Lineus viridis* (**a**) and *Lineus ruber* (**b**). The arrowhead indicates the head.

The body coloration of the studied *Lineus viridis* and *L. ruber* ranges from reddish brown to greenish brown, with the dorsal side slightly darker than the ventral side (Figure 11a,b).

**Figure 12:**
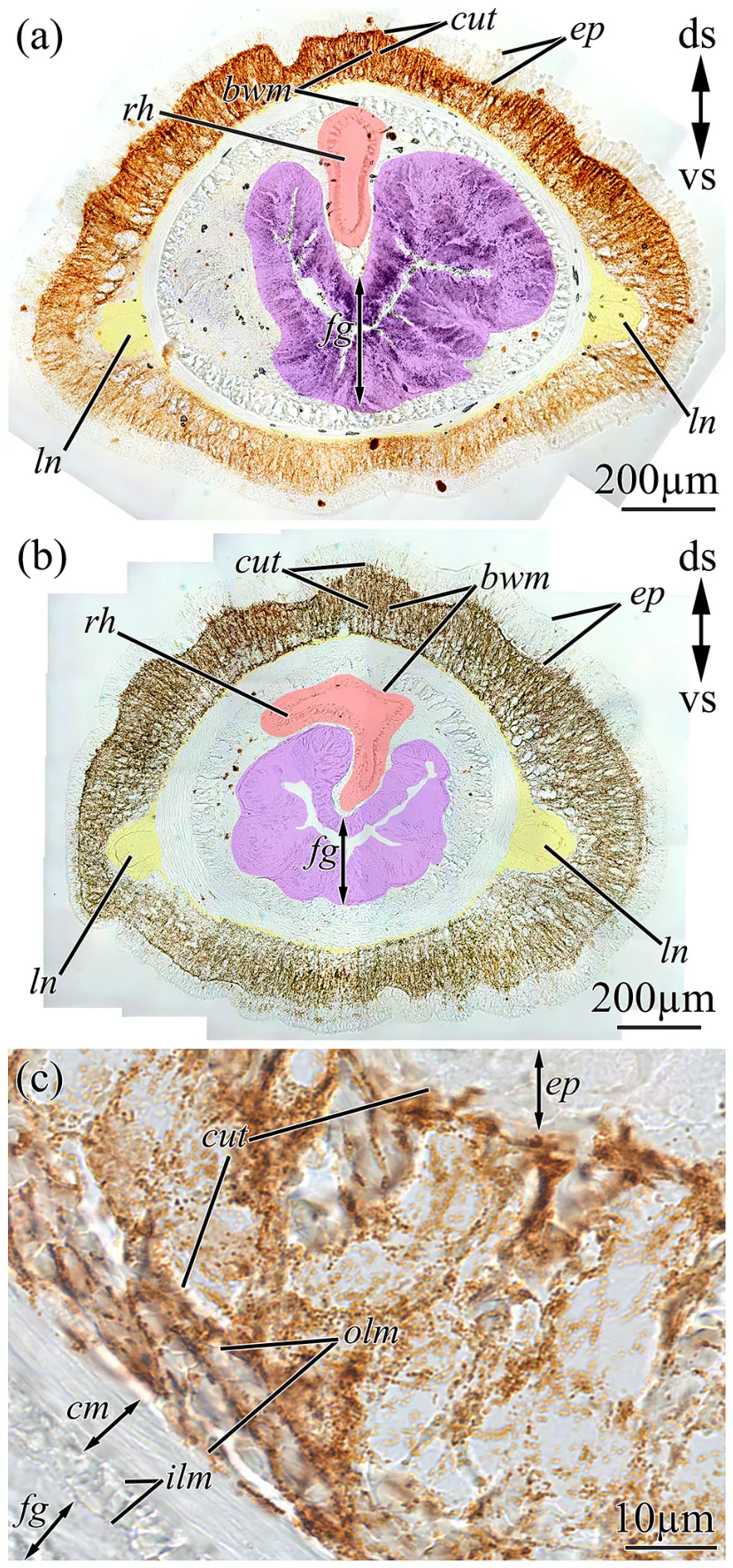
Light micrographs of thick transverse sections of the foregut region in the *Lineus ruber-viridis* species complex. (**a**) *Lineus ruber*, panoramic view of the body. (**b**) *Lineus viridis*, panoramic view of the body. (**c**) *Lineus ruber*, lateral body wall showing brown pigment granules in the cutis and outer longitudinal musculature. Abbreviations: *bwm*, body wall musculature; *cm*, circular musculature; *cut*, cutis; *ds*, dorsal side; *ep*, epidermis; *fg*, foregut; *ilm*, inner longitudinal musculature of the body wall; *ln*, lateral nerve cord; *olm*, outer longitudinal musculature of the body wall; *rh*, rhynchocoel; *vs*, ventral side.

Pigment distribution is similar in both species, regardless of differences in body coloration (Figure 12a,b). Only a single pigment type was detected (Figure 12c), represented by brown to greenish-brown granules. These granules are distributed throughout the body and occur evenly in the cutis and longitudinal musculature of the body wall, extending to the circular musculature but not penetrating it (Figure 12c).

**Figure 13:**
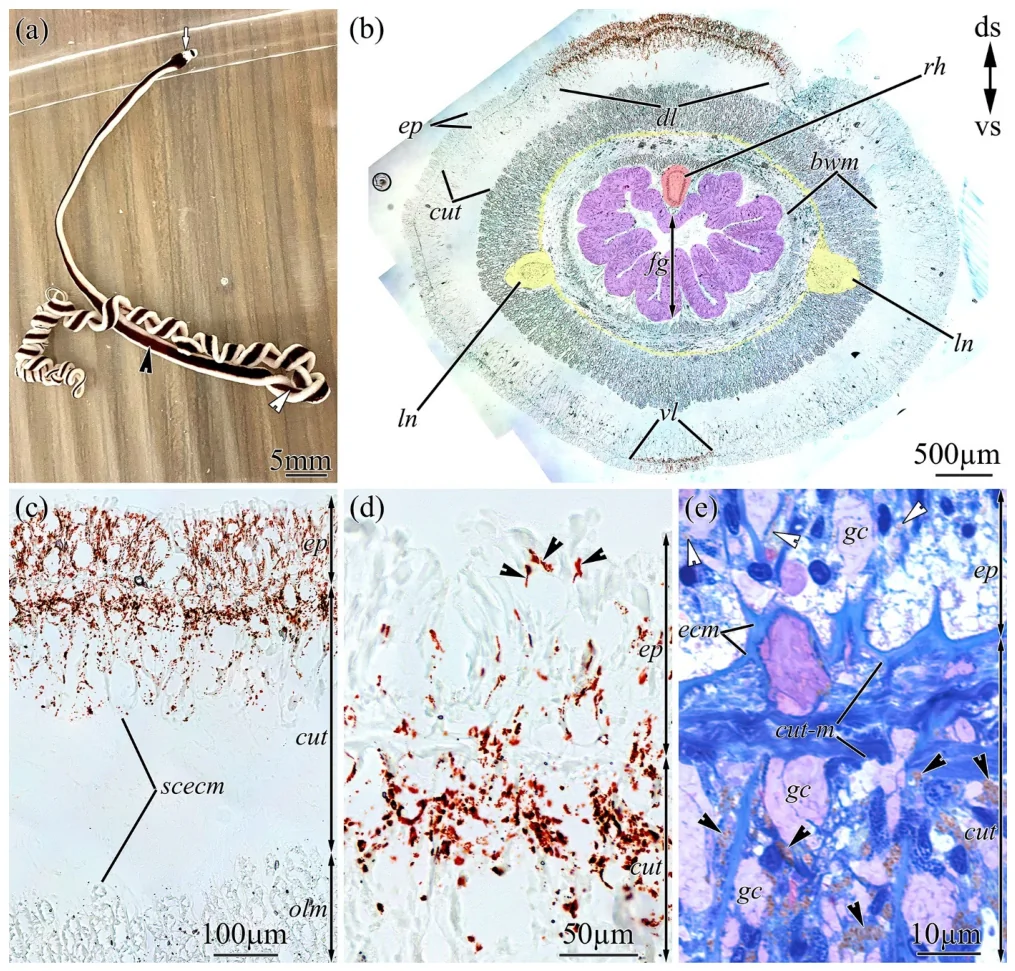
Light micrographs of a living specimen (**a**) and thick (**b**–**d**) and semithin (**e**) transverse sections of the heteronemertean *Baseodiscus hemprichii*. (**a**) Living specimen with a broad dorsal stripe (black arrowhead) and a narrow ventral stripe (white arrowhead). The white arrow indicates the head. Photograph courtesy of A.V. Chernyshev. (**b**) Panoramic view of the body in the foregut region. (**c**) Dorsal body wall. (**d**) Dorsal integument showing pigment granules (arrowheads) in the apical region of the epidermis. (**e**) Pigment granules in the basal region of the epidermis (white arrowheads) and the distal region of the cutis (black arrowheads). Abbreviations: *bwm*, body wall musculature; *cut*, cutis; *cut-m*, musculature of the cutis; *dl*, dorsal line; *ds*, dorsal side; *ecm*, extracellular matrix; *ep*, epidermis; *fg*, foregut; *gc*, glandular cell; *ln*, lateral nerve cord; *olm*, outer longitudinal musculature of the body wall; *rh*, rhynchocoel; *scecm*, subcuticular extracellular matrix; *vl*, ventral line; *vs*, ventral side.

The body of *B. hemprichii* is predominantly white (Figure 13a). The pre-cerebral region is bordered by a brown-violet stripe. Two brown-violet longitudinal stripes extend along the entire body: a wider dorsal stripe and a narrower ventral stripe. The dorsal stripe begins as a T-shaped marking near the mouth, whereas the ventral stripe begins posterior to the mouth opening.

Pigment is restricted to the regions corresponding to the dorsal and ventral stripes (Figure 13b). It occurs as brown granules (Figure 13c,d) distributed in both the cutis and the epidermis. In the upper region of the cutis, immediately beneath the cutis musculature, pigment granules form a dense accumulation (Figure 13d,e). In the epidermis, they extend to the apical region but do not contact the external environment (Figure 13d, arrowheads).

### 3.3. Ultrastructural Organization of Pigment Elements

#### 3.3.1. Ciliated Cells

Pigment was detected in the ciliated cells of *T. punctatus* and *K. torquata*.

**Figure 14:**
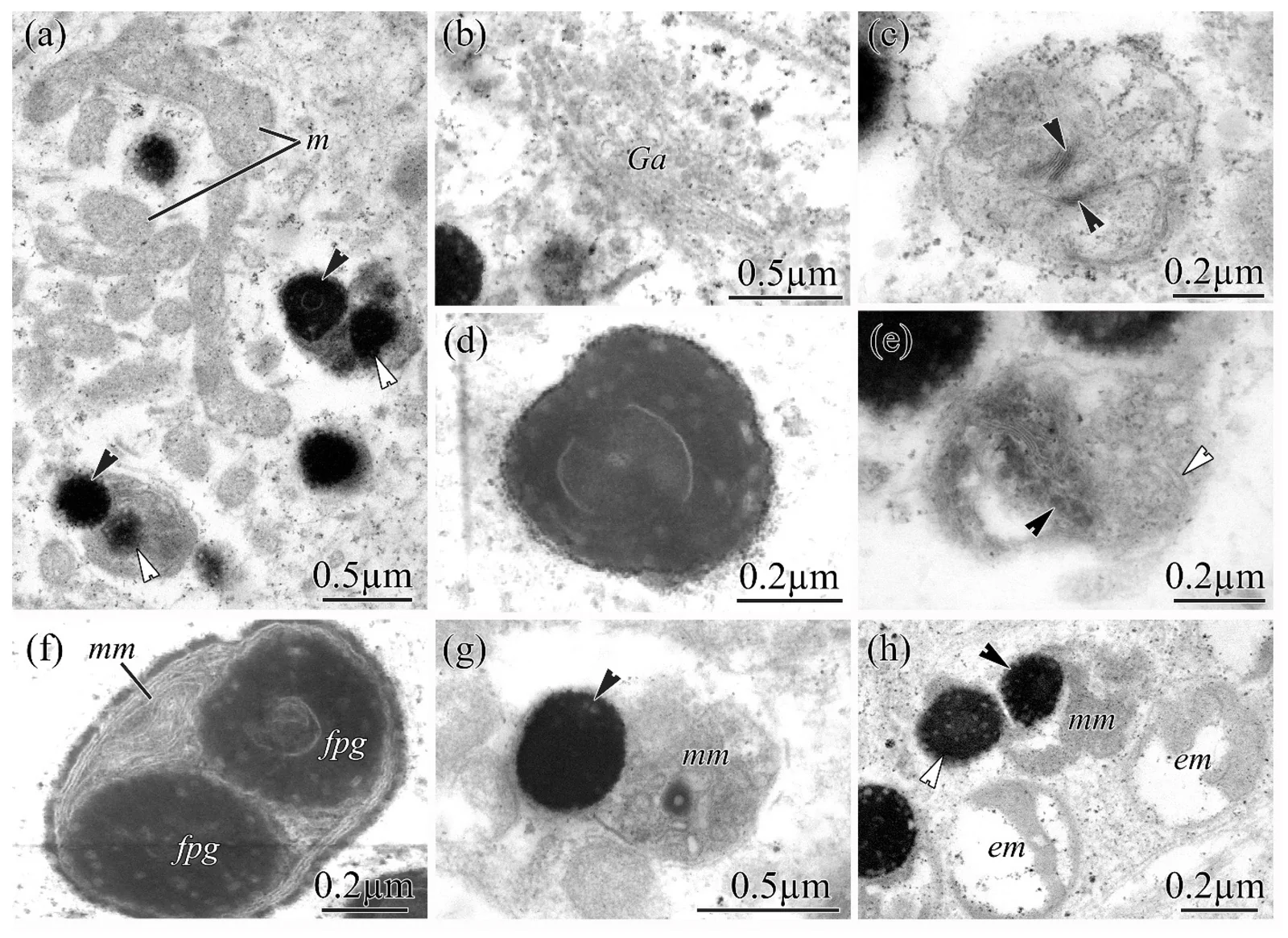
Electron micrographs of transverse sections of ciliated cells in the palaeonemertean *Tubulanus punctatus*. (**a**) Apical region showing a cluster of mitochondria and pigment granules. Black arrows indicate free pigment granules associated with modified mitochondria. White arrowheads indicate immature pigment granules within modified mitochondria. (**b**) Cell body showing a Golgi dictyosome. (**c**) Modified mitochondria with internal lamellar structures (arrowheads). (**d**) Free pigment granule. (**e**) Modified mitochondria with lamellae (white arrowhead), between which electron-dense material is located (black arrowhead). (**f**) Modified mitochondrion containing two future pigment granules. (**g**) Pigment granule associated with a modified mitochondrion (black arrow). (**h**) Middle region showing pigment granules and “empty” modified mitochondria. White arrowhead indicates a free pigment granule. Black arrowhead indicates a pigment granule associated with a modified mitochondrion. Abbreviations: *em*, empty mitochondrion; *fpg*, future pigment granule; *Ga*, Golgi apparatus; *m*, mitochondria; *mm*, modified mitochondrion.

The ciliated cells of *T. punctatus* are elongated and funnel-shaped, with a widened apical region and a narrowed basal region; the central region contains a large nucleus (Figure 1a). Mitochondria are mostly round or oval, although elongated and tortuous forms with well-defined lamellar cristae also occur; their matrix is electron-lucent (Figure 14a). They are most abundant in the apical region and sparse in the central and basal regions (Figure 14b). The Golgi apparatus (GA) is represented by a single dictyosome, located mainly in the central region of the cell (Figure 14c).

The cytoplasm of ciliated cells contains numerous spherical inclusions 0.4–0.5 µm in diameter with heterogeneous composition (pigment granules) (Figure 14b). The granules consist of a homogeneous electron-dense matrix with embedded vesicle-like structures of lower electron density (vesiculoglobular bodies) (Figure 14d). Occasionally, concentrically arranged lamellar structures are found within the pigment granules.

We have traced the stages of pigment granule maturation, which occur through structural changes in mitochondria, hereinafter termed “modified mitochondria.” Modified mitochondria are characterized by increased matrix density and the presence of elongated lamellar structures (Figure 14c). Pigment granule maturation begins with the accumulation of lamellar structures and a homogeneous electron-dense material within modified mitochondria. Electron-dense material occurs both between the lamellae and as irregularly shaped inclusions (Figure 14e). At the premature stage, these components merge into a single mass, forming a future pigment granule. At this stage, modified mitochondria contain one or two large spherical granules whose morphology is identical to that of mature pigment granules (Figure 14f). In the final stage, the granules protrude from the modified mitochondria (Figure 14a,g,h, black arrows) and are released from them (Figure 14h, white arrows). The central region of the “empty” modified mitochondria appears electron-lucent (Figure 14h).

**Figure 15:**
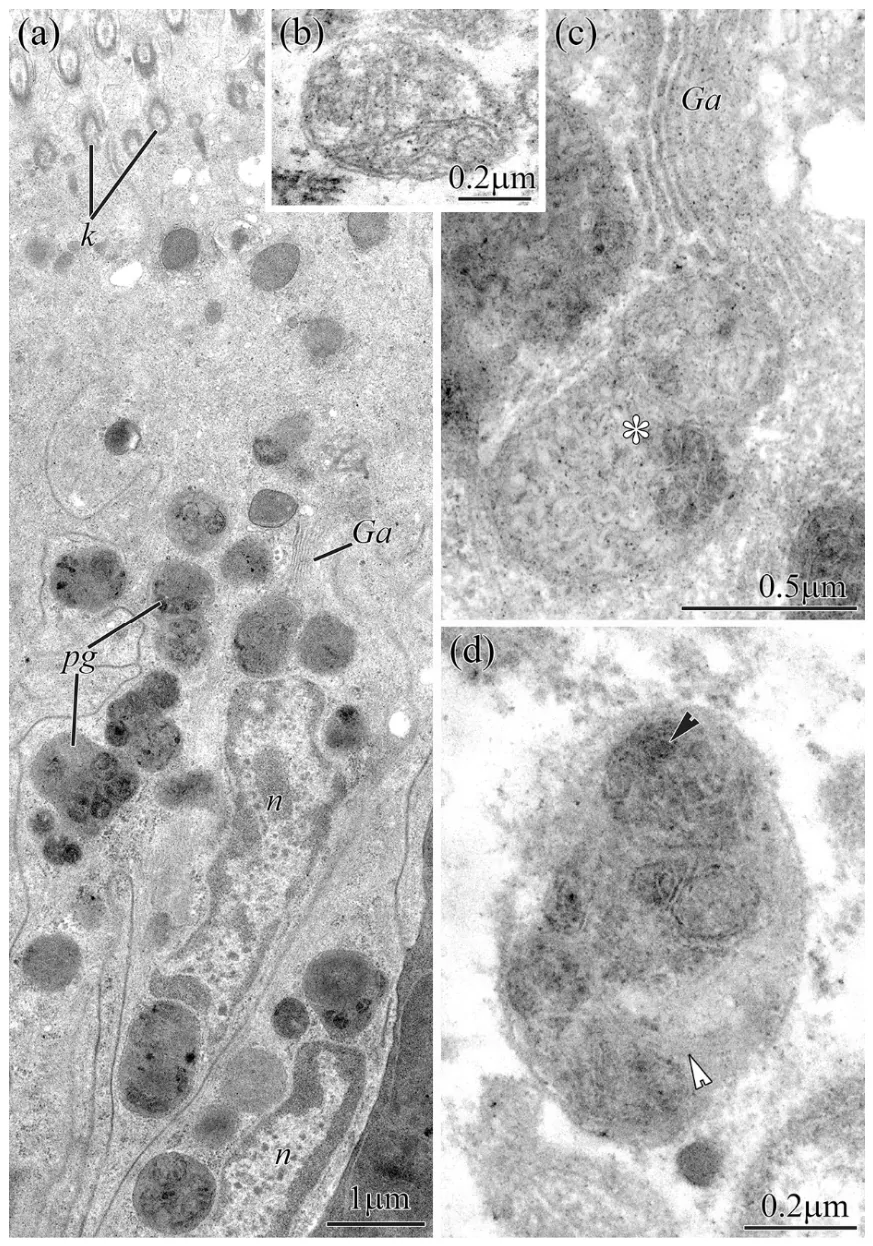
Electron micrographs of transverse sections of ciliated cells in the heteronemertean *Kulikovia torquata.* (**a**) Apical region. (**b**) Mitochondrion with well-developed cristae and electron-lucent matrix. (**c**) Perinuclear cytoplasm showing a modified mitochondrion (asterisk) associated with a Golgi dictyosome. (**d**) Mature pigment granule showing lamellar structures (white arrowhead) between which a homogeneous electron-dense material is located (black arrowhead). Abbreviations: *Ga*, Golgi apparatus; *k*, kinetosome; *n*, nucleus; *pg*, pigment granule.

The ciliated cells of *K. torquata* have the same funnel-shaped form as those of *T. punctatus* but differ in the position of the nucleus and GA, and in the distribution of mitochondria and pigment granules. The nucleus is located in the apical-central region of the cell (Figure 15a). Mitochondria are round or oval, with well-defined lamellar cristae, and are evenly distributed without forming clusters (Figure 15b). The GA comprises one or two dictyosomes, located primarily above the nucleus. Numerous pigment granules are concentrated in the apical region of the cell, mainly near the GA, with a smaller proportion in the perinuclear cytoplasm (Figure 15a,c). Observations show that pigment granules are derived from modified mitochondria. At early stages of maturation, these mitochondria are characterized by increased matrix density and expanded, wave-profiling cristae (Figure 15c). Electron-dense material appears between the cristae (lamellae) and progressively accumulates, partially replacing the matrix, until the entire mitochondrion is transformed into a pigment granule (Figure 15d).

#### 3.3.2. Pigment Cells

**Figure 16:**
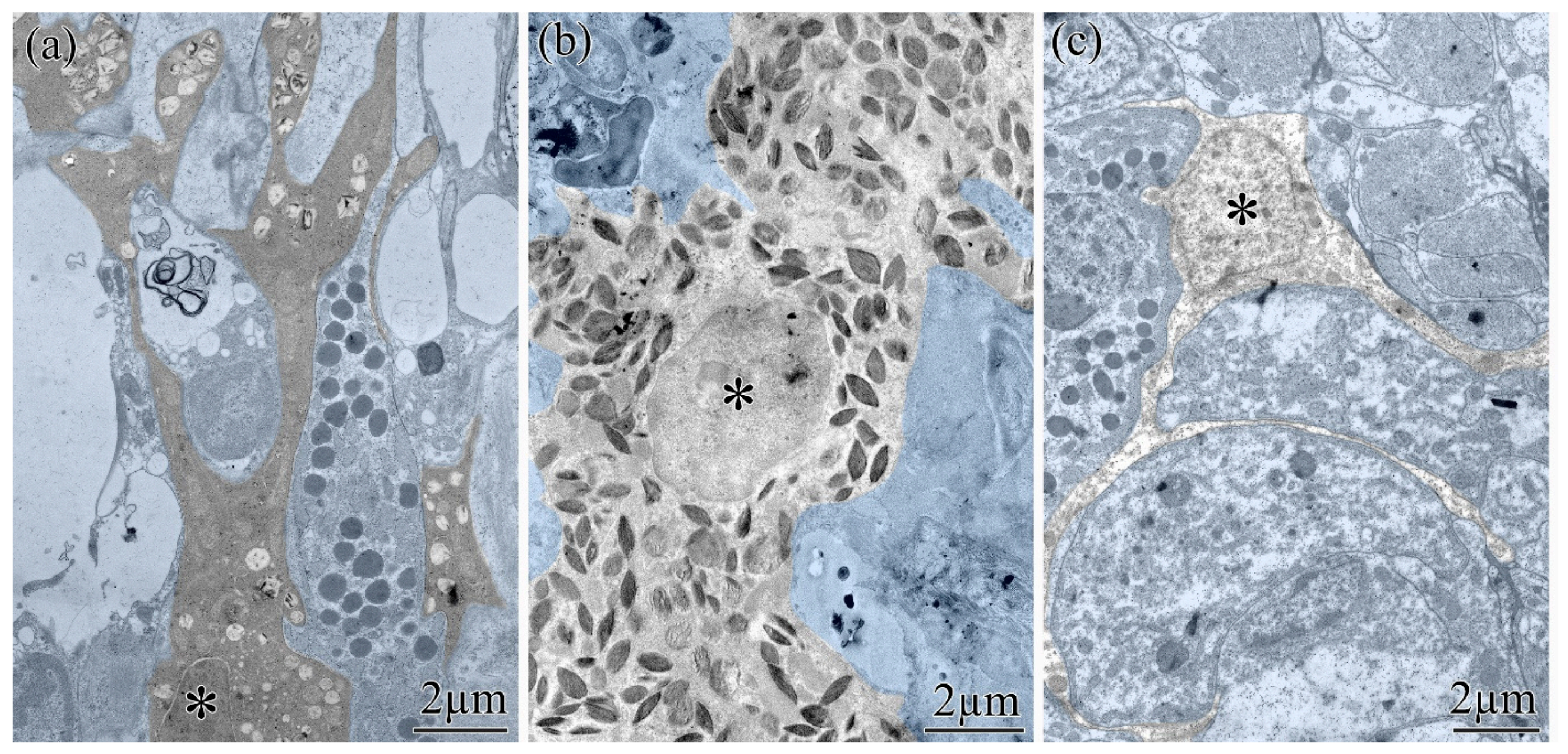
Electron micrographs of transverse sections of pigment cells in selected nemerteans. Blue color marks tissue surrounding pigment cells (**a**) *Oerstedia oculata*, pigment cell (asterisk) in the middle region of the epidermis. (**b**) *Tetrastemma stimpsoni*, pigment cell (asterisk) in the basal region of the epidermis. (**c**) *Lineus viridis*, pigment cell (asterisk) in the cutis.

In the integument of the studied hoplonemerteans and heteronemerteans (except *K. torquata*), a distinct cell type, pigment cells, is present. These cells contain uniform pigment granules that likely determine the macroscopic body coloration; no pigment granules were observed outside these cells. Pigment cells are amoeboid, with numerous thin, highly branched cytoplasmic processes extending from the cell body (Figure 16a,c). Their nuclei are large and euchromatic. The cytoplasm is light in *Te. stimpsoni*, *K. manchenkoi*, *K. alborostrata* and *B. hemprichii* (Figure 16b), dark in *O. oculata* (Figure 16a), and granular in *Lineus viridis* and *L. ruber*. (Figure 16c). It contains endoplasmic reticulum (ER), one to two Golgi dictyosomes, and mitochondria.

**Figure 17:**
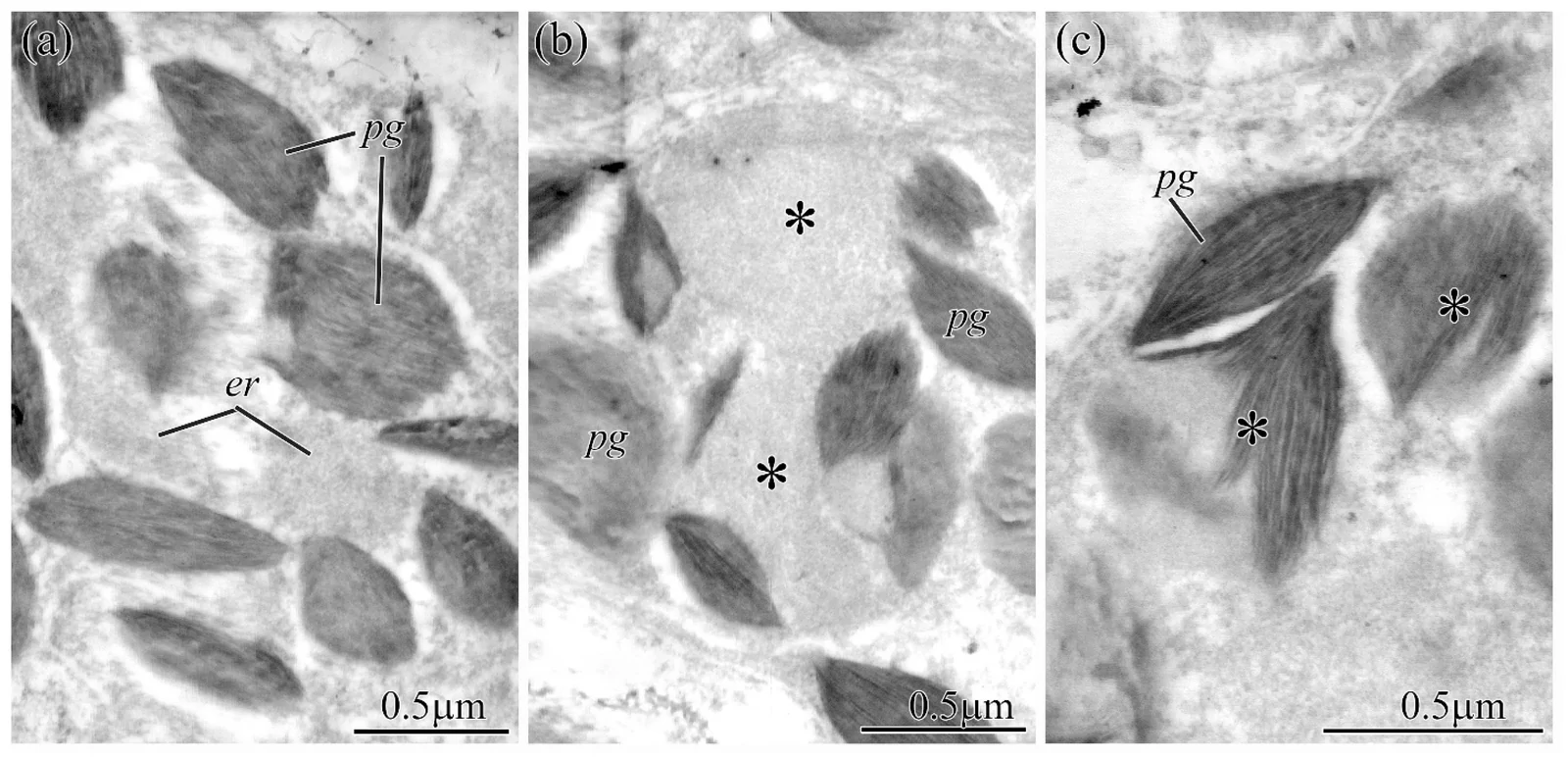
Electron micrographs of transverse sections of the perinuclear cytoplasm of a pigment cell of the hoplonemertean *Tetrastemma stimpsoni*. (**a**) Dilated cisternae of the endoplasmic reticulum located between mature pigment granules. (**b**) Immature pigment granules (asterisks). (**c**) Premature pigment granules (asterisks). Abbreviations: *er*, endoplasmic reticulum; *pg*, pigment granule.

In *Te. stimpsoni*, pigment cells show the same ultrastructural organization in both uniformly dark and striped morphs, but are absent in the pale-yellow morph. The cell bodies are located mainly in the basal region of the epidermis, resting on the cup layer. Cytoplasmic processes extend along the cup layer and toward the epidermal surface, but do not reach it. Pigment granules are spindle-shaped, measuring 0.2–0.4 µm in diameter and up to 0.8 µm in length (Figure 17a,c). The granules have a heterogeneous content: electron-dense fibrils (up to 10 nm thick), arranged in parallel and converging at the poles, are embedded in an electron-lucent homogeneous matrix. Pigment granules originate from ER cisternae, which are initially dilated and filled with finely granular material of moderate electron density (Figure 17a). These structures subsequently become rounded and develop into immature pigment granules with heterogeneous contents, comprising electron-dense regions of homogeneous material embedded within a finely granular matrix (Figure 17b). At the final stage of maturation, fibrils form at one pole, radiating from the apex in a fan-like pattern (Figure 17c).

**Figure 18:**
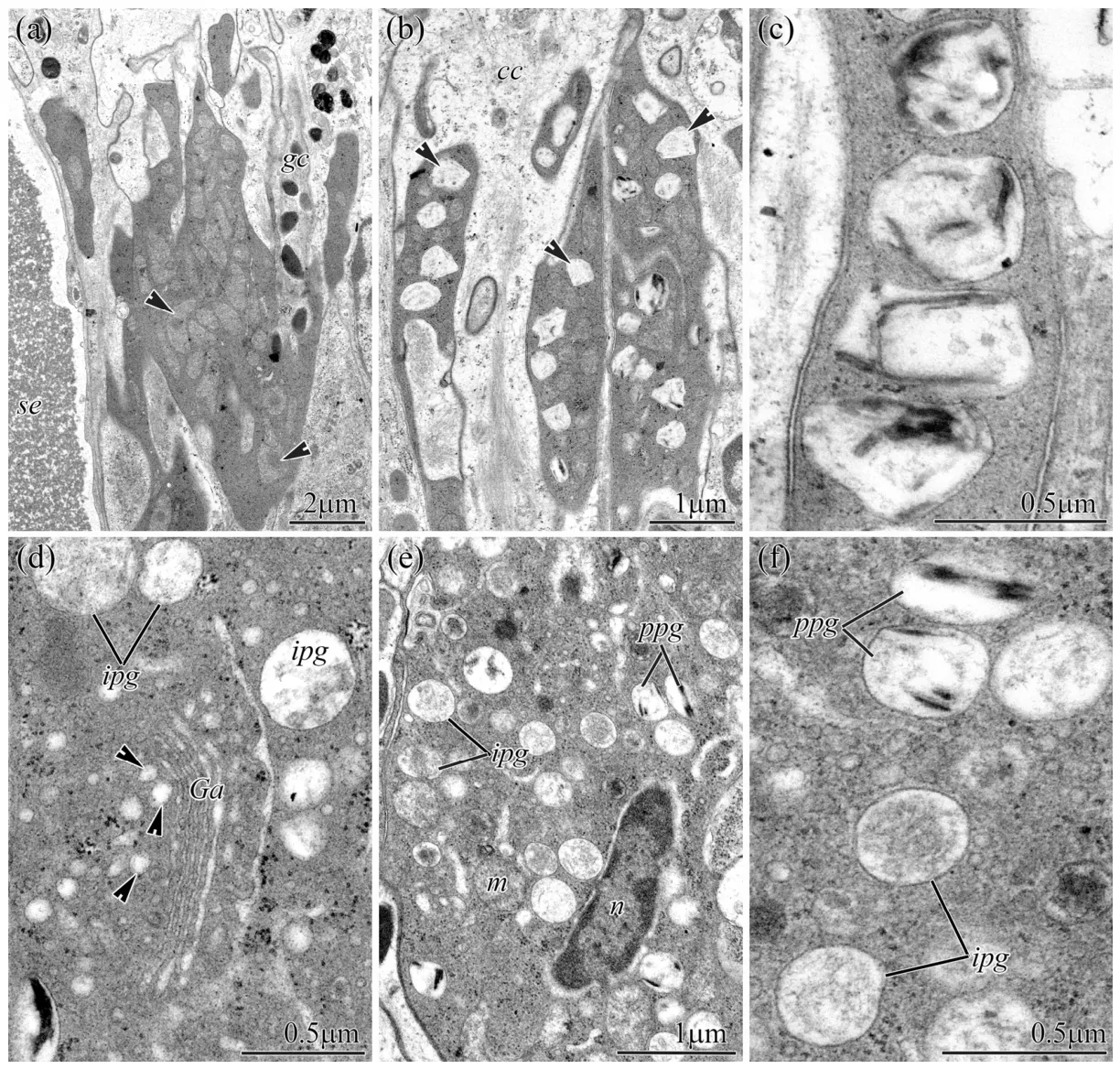
Electron micrographs of transverse sections of pigment cells of the hoplonemertean *Oerstedia oculata.* (**a**) Central region of the ventral epidermis showing pigment cell processes containing vacuoles (arrowheads). (**b**) Central region of the dorsal epidermis showing pigment cell processes containing pigment granules (arrowheads). (**c**) Pigment cell process filled with pigment granules. (**d**) Perinuclear cytoplasm of a pigment cell showing the Golgi apparatus associated with vesicles (arrowheads) and immature pigment granules. (**e**) Cell body of a pigment cell showing immature and premature pigment granules. (**f**) Perinuclear cytoplasm of a pigment cell containing immature and premature pigment granules. Abbreviations: *cc*, ciliated cell; *gc*, glandular cell; *Ga*, Golgi apparatus; *ipg*, immature pigment granule; *m*, mitochondria; *n*, nucleus; *ppg*, premature pigment granule; *se*, serous cell.

In *O. oculata*, pigment cells are found on both the dorsal and ventral sides of the body (Figure 18a,b). Their cell bodies are located in the basal and central regions of the epidermis. Most cytoplasmic processes extend toward the epidermal surface, with one or two directed toward the cup layer (Figure 16a). Pigment cells contain two types of inclusions: vacuoles and pigment granules. Vacuoles are elongated-oval (about 0.5 µm in diameter and up to 1.5 µm in length) and contain finely granular material of moderate electron density (Figure 18a). They are present in pigment cells on both the dorsal and ventral sides. Pigment granules are rounded with angular edges (0.3–0.6 µm in diameter) and contain an electron-lucent matrix with polygonal and/or rod-shaped electron-dense inclusions (Figure 18c). These granules occur only on the dorsal and lateral sides and are absent on the ventral side (Figure 18b). Pigment granules originate in the cell body from the GA, with small vesicles (about 0.1 µm in diameter) containing electron-lucent material budding from the cis-face (Figure 18d). Near the GA and in the perinuclear cytoplasm, immature pigment granules (0.2–0.6 µm in diameter) contain flocculent or loosely to moderately packed fibrillar–granular material (Figure 18e). In the premature pigment granules, this material condenses into thick, dense rod-shaped structures (Figure 18f). At the final stage, vesicles change their shape and the number of rod-shaped structures increases, forming mature pigment granules.

**Figure 19:**
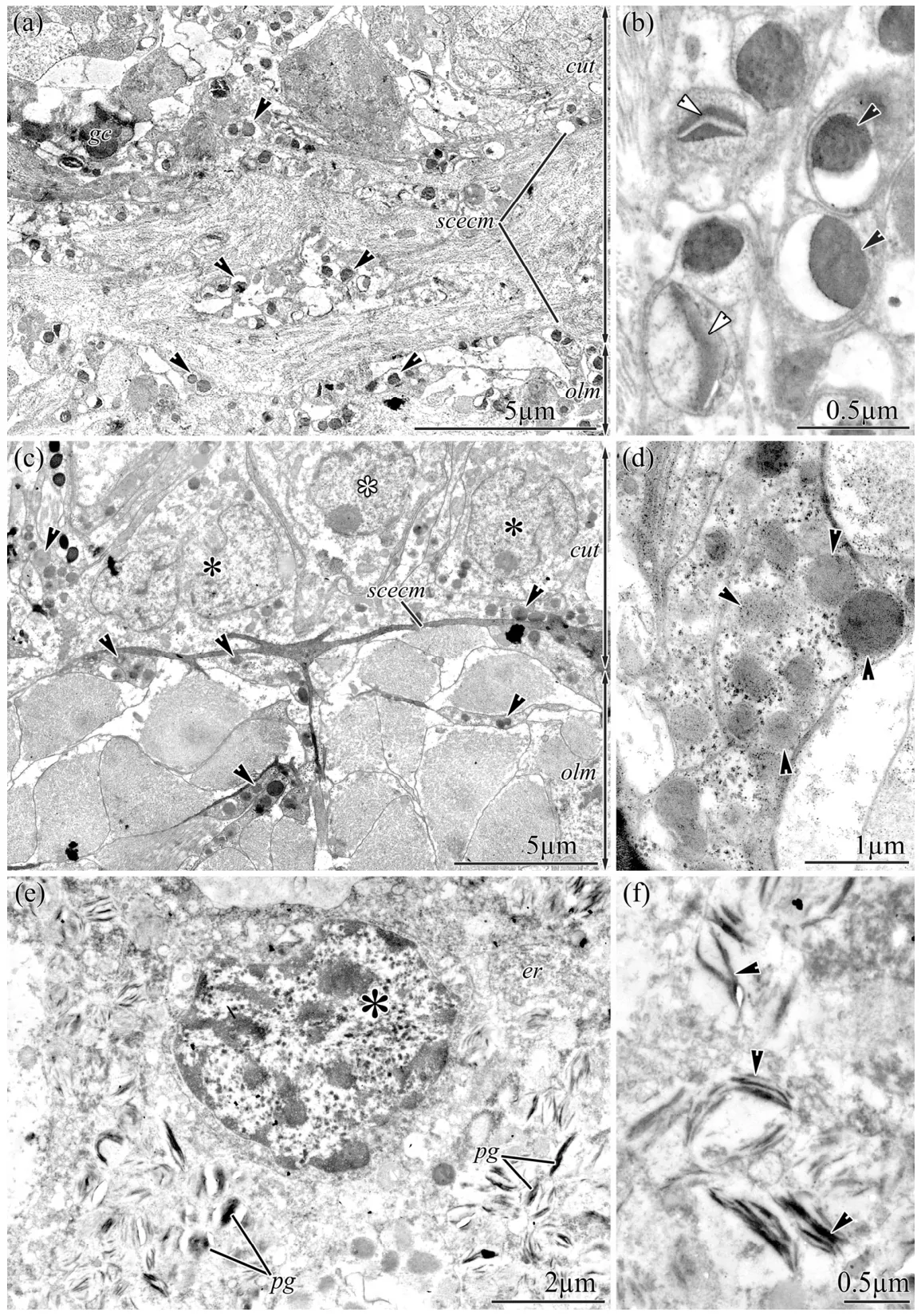
Electron micrographs of transverse sections of selected heteronemerteans. (**a**) *Kulikovia manchenkoi*, pigment cell processes (arrowheads) located in the proximal region of the cutis, subcutis extracellular matrix (ECM), and outer longitudinal musculature of the body wall. (**b**) *K. manchenkoi*, pigment cell processes showing pigment granules with round (black arrowheads) and crescent-shaped (white arrowheads) inclusions. (**c**) *Lineus viridis*, pigment cell processes (arrowheads) located in the proximal region of the cutis, subcutis ECM, and outer longitudinal musculature of the body wall. Black asterisks indicate pigment cell bodies. A white asterisk indicates a glandular cell body. (**d**) *L. viridis*, pigment cell process filled with pigment granules (arrowheads). (**e**) *Baseodiscus hemprichii*, pigment cell body (asterisk) located in the cutis. (**f**) *B. hemprichii*, pigment cell process filled with pigment granules (arrowheads). Abbreviations: *cut*, cutis; *ecm*, extracellular matrix; *er*, endoplasmic reticulum; *gc*, glandular cell; *olm*, outer longitudinal musculature of the body wall; *pg*, pigment granule; *scecm*, subcutis extracellular matrix.

In all studied heteronemerteans, the cell bodies of pigment cells are located exclusively in the cutis. In all species except *B. hemprichii*, cytoplasmic processes occur in the cutis and the longitudinal musculature of the body wall (Figure 19a,c). In *B. hemprichii*, they are confined to the cutis and epidermis.

In the dark morphs of *K. manchenkoi* and *K. alborostrata*, pigment cells are found on both the dorsal and ventral sides, but are absent in light morphs. In both species, pigment granules share the same ultrastructural organization: they are spherical (0.3–0.4 µm in diameter) and contain heterogeneous contents, with electron-dense round or crescent-shaped material displaced toward one pole and embedded in a loosely fibrillar matrix (Figure 19b).

Pigment cells of *L. ruber* and *L. viridis* contain granules of similar organization: spherical (0.3–0.5 µm in diameter) and filled with homogeneous material of moderate electron density (Figure 19d).

In *B. hemprichii*, pigment cells were identified at the ultrastructural level only in brown-violet stripes. The granules are spherical (0.7–0.9 µm in diameter) and contain thick fibrils (about 25 nm in diameter), straight or slightly curved and arranged in parallel but not closely packed, embedded in a homogeneous matrix of low electron density (Figure 19e,f).

#### 3.3.3. Leucophores

**Figure 20:**
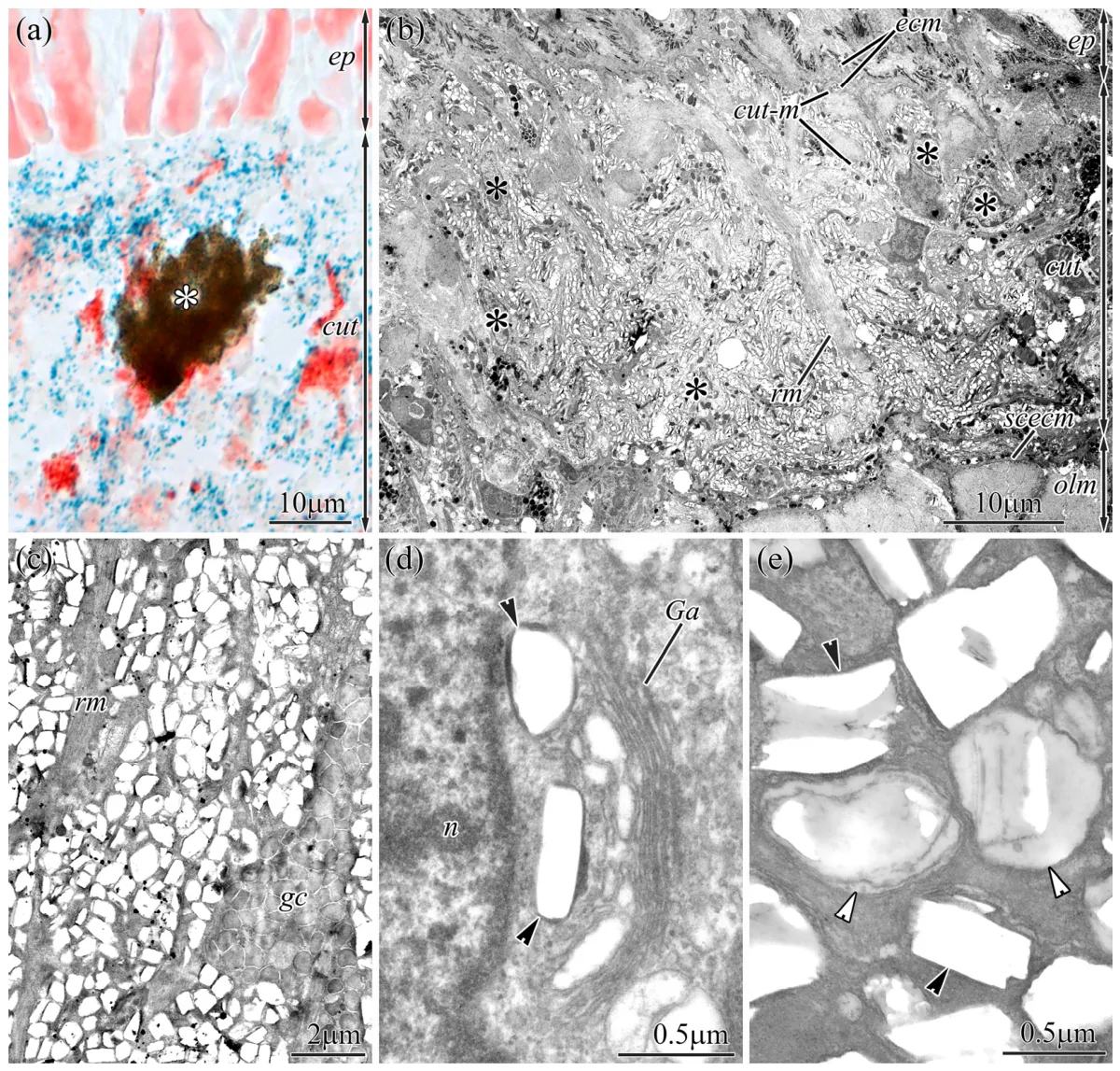
Light (**a**) and electron micrographs (**b**–**e**) of transverse sections of the body wall in regions of white spots in *Kulikovia manchenkoi* (**a**,**c**,**e**) and *Kulikovia torquata* (**b**,**d**). (**a**) Distal region of the cutis showing an accumulation of leucophores (asterisk). (**b**) Overview of leucophores (asterisks) in the cutis. (**c**) Cytoplasmic processes of leucophores located between radial musculature and glandular cells of the cutis. (**d**) Perinuclear cytoplasm of a leucophore showing the Golgi apparatus associated with reflector plates (arrowheads). (**e**) Cytoplasmic process of a leucophore containing reflector plates of round (white arrowheads) and rectangular (black arrowheads) shapes. Abbreviations: *cut*, cutis; *ecm*, extracellular matrix; *ep*, epidermis; *gc*, glandular cell; *Ga*, Golgi apparatus; *n*, nucleus; *olm*, outer longitudinal musculature of the body wall; *rm*, radial musculature; *scecm*, subcutis extracellular matrix.

In the cutis of *K. manchenko* and *K. torquata*, unstained light micrographs reveal brown clusters (Figure 20a) corresponding to white specks visible on the body surface (Figure 8a,b and Figure 10a). At the ultrastructural level, these spots are formed by accumulations of leucophores. Leucophores are located between cutis components, including cutis musculature, radial musculature, glandular cells, and pigment cells (Figure 20b,c). The cells have large, elongated nuclei. In the perinuclear cytoplasm, one or two Golgi dictyosomes are present, each comprising five to eight cisternae that are flattened at the cis pole and dilated at the trans pole, where they contain homogeneous material of low electron density (Figure 20d). Most of the cytoplasm is occupied by granules (reflector plates) arranged irregularly (Figure 20c). Reflector plates are mostly rectangular or rhomboid, with facet dimensions of about 0.2–0.6 µm (Figure 20e). Their electron-lucent appearance likely reflects a fixation artifact. Some granules are round (about 0.6 µm in diameter) and contain homogeneous material of low electron density with distinct polygonal inclusions (Figure 20e).

## 4. Discussion

**Figure 21:**
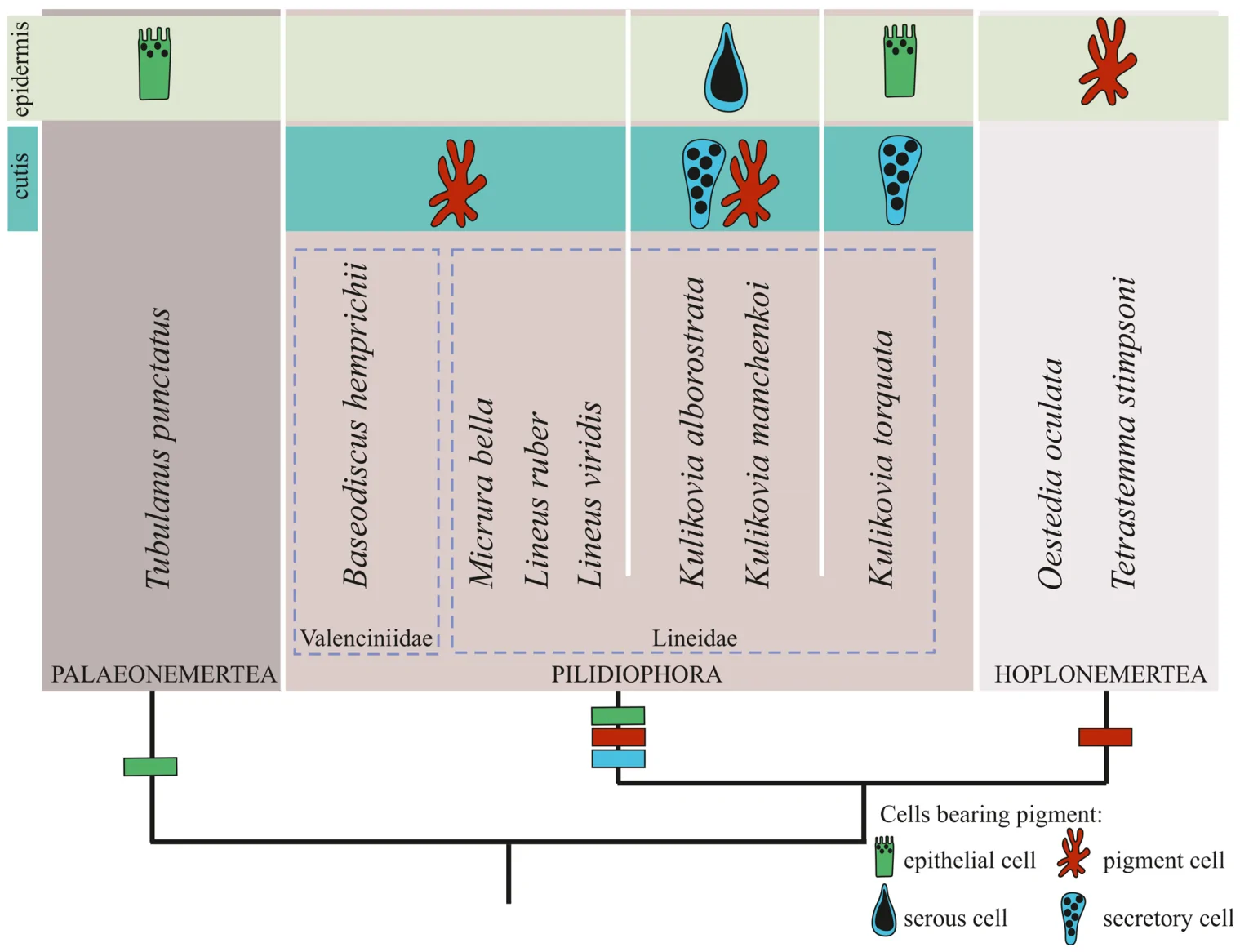
Distribution of pigment cells in nemerteans. Color map: blue, glandular cells; green, epidermal cells; red, pigment cells.

Our study demonstrates that the distribution of pigment-bearing cells in nemerteans is heterogeneous and varies significantly among taxa (Figure 21). This variability is most pronounced in heteronemerteans, where both closely and distantly related species were examined. *Kulikovia alborostrata* and *K. manchenkoi* show similar patterns of pigment distribution: in light morphs, pigment is confined to glandular cells of the cutis and epidermis, whereas in dark morphs it also occurs in pigment cells of the cutis. In contrast, *K. torquata*, a species closely related to *K. manchenkoi*, exhibits a different pattern, with pigment present in glandular cells of the cutis and in ciliated cells. Chernyshev et al. [14] previously reported differences in the cellular composition of cutis glandular cells between these cryptic species; our data indicate that pigment distribution may serve as an additional diagnostic character. By comparison, the in *Lineus viridis* and *L. ruber* examined here show no variation in either the cellular composition or the localization of pigment cells. In these species, body coloration is determined by pigment cells located in the cutis and the outer longitudinal musculature of the body wall. A similar organization has been described for closely related taxa; for example, in *Lineus sanguineus*, pigment cells occur in the same regions and contain granules of comparable ultrastructure [15]. The limited sampling of hoplonemerteans and palaeonemerteans in this study does not allow general conclusions about pigment organization in these groups. However, the pronounced variability observed in heteronemerteans suggests that pigment distribution in these taxa may be more diverse than currently recognized.

Pigment cells of nemerteans show an amoeboid morphology typical of chromatophores, making them comparable to those of vertebrates (fishes, amphibians, reptiles) and cephalopods [16]. In *K. torquata* and *K. manchenkoi*, leucophores were also observed alongside pigment cells. Their ultrastructure corresponds to that described in other taxa [17,18] and is consistent with structural light reflection rather than pigment-based coloration [19,20].

Our results suggest that pigment expression in nemerteans follows two distinct patterns. In the first, pigment cells consistently contain pigment and directly determine body coloration. This occurs in *Te. stimpsoni*, *K. alborostrata*, *K. manchenkoi*, *Lineus viridis*, *L. ruber* and *B. hemprichii*. In the second, pigment cells are uniformly distributed throughout the body, but pigment production is restricted to a subset of cells, so coloration depends on localized pigment synthesis. This pattern is observed in *O. oculata* and *M. bella* ([8]; present study). In the former case, color patterning is determined by the spatial distribution of pigment cells during development, as is typical for many animals [21,22,23,24]. By contrast, the second pattern implies that pigmentation is regulated at the level of cell activity rather than cell positioning. A uniform distribution of pigment cells with restricted pigment production has not been reported previously. However, functionally inactive melanocytes—cells lacking mature melanosomes that coexist with actively melanogenic cells—are known in vertebrates [25]. This pattern may be controlled by morphogenetic or local regulatory signals that trigger pigment synthesis in selected cells. The occurrence of both patterns in closely related species indicates a degree of plasticity in pigment cell organization and its regulation in nemerteans. This pattern may be controlled by morphogenetic or local regulatory signals that activate pigment synthesis in selected cells. The occurrence of both patterns in closely related species indicates a degree of plasticity in pigment cell organization and its regulation in nemerteans.

**Figure 22:**
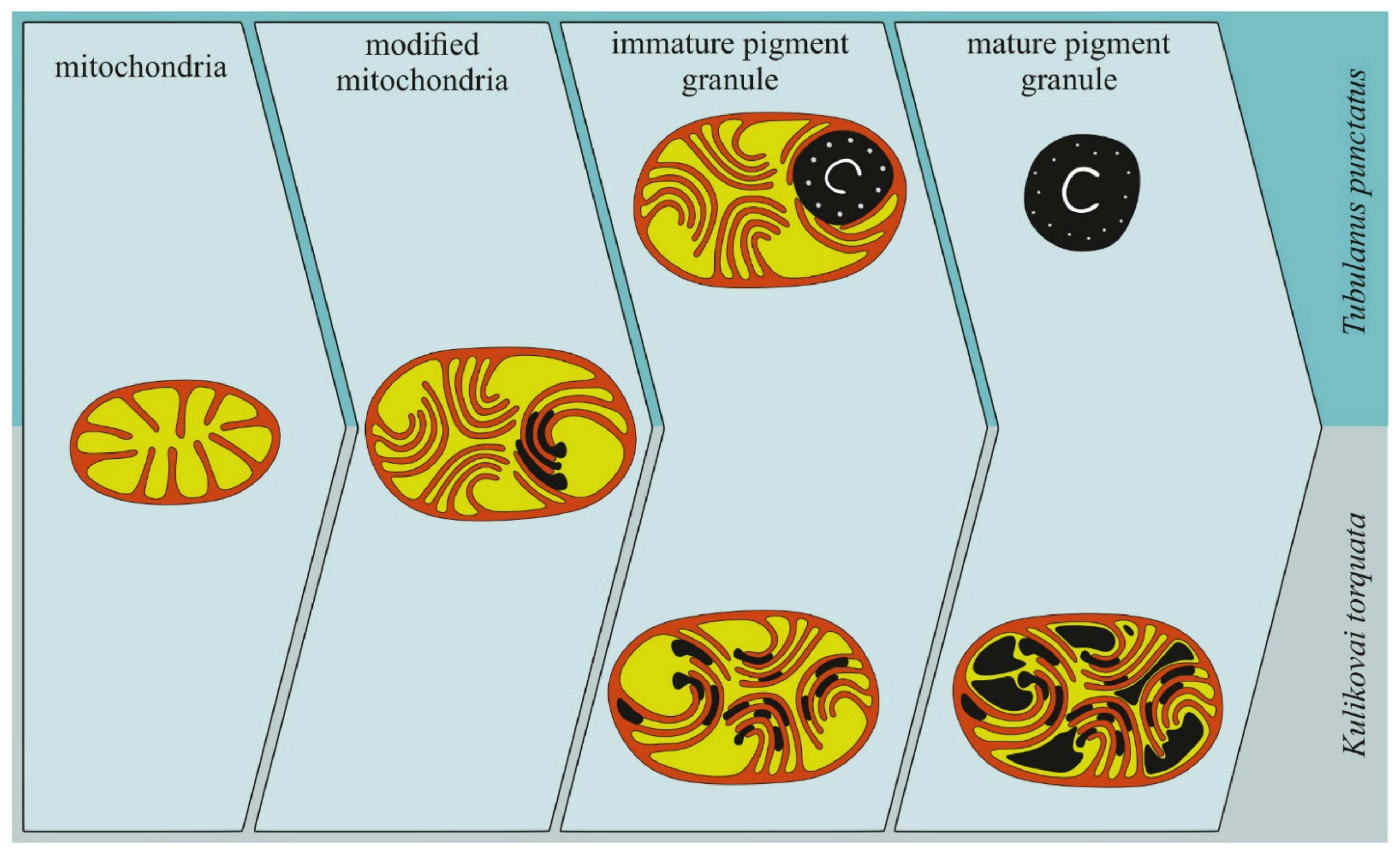
Schematic representation of pigment granule maturation from mitochondria in the nemerteans *Tubulanus punctatus* and *Kulikovia torquata*. Color map: black, pigment; orange, mitochondrial cristae and membrane; yellow, mitochondrial matrix.

In *T. punctatus* and *K. torquata*, pigment is found in ciliated epithelial cells (Figure 21). To our knowledge, no comparable pattern of pigment localization has been described in other animals. A possible exception is the epithelial lining of the ink sac in *Sepia officinalis* [26]; however, in this case pigment is associated with the specialized function of a secretory organ rather than with general integumentary coloration. The presence of pigment in epithelial cells suggests that the pigment system of nemerteans may have evolved differently from that of most bilaterians, in which pigment is typically confined to specialized chromatophores. Consistent with this, in basally branching flatworms, pigment is associated with specialized cells located within the parenchyma [27,28]. In nemerteans, pigment formation in ciliated cells occurs within mitochondria: pigment accumulates in the matrix between the cristae and either remains within the organelle, forming a granule (*K. torquata*), or is released as a mature granule (*T. punctatus*) (Figure 22). This mechanism differs from previously described pigmentogenic pathways, which involve the endoplasmic reticulum and/or GA [29]. However, mitochondria are known to play an indirect regulatory role in melanogenesis beyond energy production [30,31,32]. Our observations suggest that mitochondria in nemerteans may retain an ancestral role in the direct formation of pigment granules. Biochemical and molecular genetic studies are required to test this hypothesis.

## 5. Conclusions

In the present study, we show that the cellular basis of body coloration in nemerteans is heterogeneous and involves not only specialized chromatophores but also glandular and ciliated epithelial cells. Pigment systems within Nemertea are diverse, with heteronemerteans exhibiting the greatest diversity of pigment structures, likely reflecting the increased structural complexity of their integument associated with the development of a cutis. In contrast, palaeo- and hoplonemerteans show a more uniform organization, in which a single cell type contributes to coloration. At the subcellular level, pigment formation pathways also differ among cell types: in ciliated epithelial cells, granule formation is associated with mitochondria, whereas in chromatophores it is linked to the endoplasmic reticulum and/or GA. However, palaeo- and hoplonemerteans remain insufficiently represented in the present morphological dataset, which highlights the need for further investigation. Despite the diversity of coloration mechanisms identified here, the chemical nature of nemertean pigments remains poorly understood. Future studies should therefore focus on their chemical characterization, which is essential for understanding their origin, evolutionary plasticity, and physiological roles.

## Figures and Tables

**Table 1:** GenBank accession numbers for cytochrome c oxidase subunit I sequences of nemertean species analyzed in this study.

Species	GenBank Accession Number	Reference
*Tubulanus punctatus*	PZ610258	Current study
*Oerstedia oculata*	MN211495	[11]
*Tetrastemma stimpsoni*	PZ571182	Current study
*Micrura bella*	OQ450494	[12]
*Kulikovia alborostrata*	OR883925	[13]
*Kulikovia manchenkoi*	PX463917	Current study
*Kulikovia torquata*	OR883927	Current study
*Lineus ruber*	PZ477597	Current study
*Lineus viridis*	PZ610259	Current study

## Data Availability

The original images are available at https://doi.org/10.6084/m9.figshare.32928917.

## Abbreviations

The following abbreviations are used in this manuscript:

CB	Sodium cacodylate buffer
ECM	Extracellular matrix
ER	Endoplasmic reticulum
GA	Golgi apparatus
PBS	Phosphate-buffered saline
PCR	Polymerase Chain Reaction
